# The AI Economist: Taxation policy design via two-level deep multiagent reinforcement learning

**DOI:** 10.1126/sciadv.abk2607

**Published:** 2022-05-04

**Authors:** Stephan Zheng, Alexander Trott, Sunil Srinivasa, David C. Parkes, Richard Socher

**Affiliations:** 1 Salesforce Research, Palo Alto, CA, USA.; 2 Harvard University, Cambridge, MA, USA.; 3 You.com, Palo Alto, CA, USA.

## Abstract

Artificial intelligence (AI) and reinforcement learning (RL) have improved many areas but are not yet widely adopted in economic policy design, mechanism design, or economics at large. The AI Economist is a two-level, deep RL framework for policy design in which agents and a social planner coadapt. In particular, the AI Economist uses structured curriculum learning to stabilize the challenging two-level, coadaptive learning problem. We validate this framework in the domain of taxation. In one-step economies, the AI Economist recovers the optimal tax policy of economic theory. In spatiotemporal economies, the AI Economist substantially improves both utilitarian social welfare and the trade-off between equality and productivity over baselines. It does so despite emergent tax-gaming strategies while accounting for emergent labor specialization, agent interactions, and behavioral change. These results demonstrate that two-level, deep RL complements economic theory and unlocks an AI-based approach to designing and understanding economic policy.

## Teaser

The AI Economist finds tax policies that yield higher social welfare compared to progressive, regressive, or no taxes.

## INTRODUCTION

Economic policies need to be optimized to tackle critical global socioeconomic issues and achieve social objectives. For example, tax policy needs to balance equality and productivity, as large inequality gaps cause loss of economic opportunity (*1*) and adverse health effects (*2*). However, the problem of optimal policy design is very challenging, even when the policy objectives can be agreed upon.

Policy optimization poses a mechanism design (*3*) problem: The government (social planner) aims to find a policy under which the (boundedly) rational behaviors of affected economic agents yield the desired social outcome. We only consider rational behaviors in this work, although our framework can be extended to include boundedly rational actors. Using rational agents serves as an important benchmark (*4*). Moreover, the optimal taxation problem satisfies the two conditions for rationality to be a good assumption: It is high stakes, and it is a repeated setting (*5*).

Theoretical approaches to policy design are limited by analytical tractability and thus fail to capture the complexity of the real world. Empirical studies are challenged by the lack of counterfactual data and face the Lucas critique (*6*) that historical data do not capture behavioral responses to policy behavior. Furthermore, opportunities for rigorous, real-world experimentation are limited and come with ethical questions (*7*).

Computational and machine learning techniques for automated mechanism design (*8*–*12*) show promise toward overcoming existing limitations, but there is no general computational framework for policy design. The challenge with policy design comes from needing to solve a highly nonstationary, two-level, sequential decision-making problem where all actors (the agents and the government) are learning: Economic agents learn rational, utility-maximizing behaviors, and the government learns to optimize its own objective via policy choices.

### A new machine learning challenge

Using deep reinforcement learning (RL) with multiple agents has been underexplored as a solution framework for mechanism design. Recent advances in deep RL have mostly studied the single-level setting; for example, state-of-the-art deep RL systems such as AlphaGo (*13*) and AlphaStar (*14*) optimized actors under fixed reward functions. In contrast, in the two-level setting, agents’ effective reward functions depend on (changes in) the planner’s policy, which leads to a highly unstable learning and coadaptation problem.

Advances in multiagent RL have focused on cooperative problems in complex spatiotemporal games (*14*, *15*) and social dilemmas with fixed reward functions (*16*). However, multiagent deep RL has not been used to design government policies in large economies, which can be modeled as dynamical systems with a large number of heterogeneous self-interested RL agents with changing incentives. More generally, economics as a field has not seen wide adoption of deep RL or related artificial intelligence (AI) methods.

### The AI Economist

We introduce the AI Economist, a framework that combines machine learning and AI-driven economic simulations to overcome the limitations faced by existing approaches. The AI Economist shows the efficacy and viability of using two-level RL as a new paradigm for economic policy design.

We show that two-level RL can find policies that yield higher social welfare compared with standard baselines. Moreover, we show that the learned behaviors of AI agents in economic simulations can resemble real-world economic phenomena such as worker specialization. Hence, AI-driven economic simulations can be useful without the need for human-coded, application-specific rules.

### AI-driven simulations

We use both a single-step economy and Gather-Trade-Build, a multistep, microfounded economic simulation. Gather-Trade-Build features multiple heterogeneous economic agents in a two-dimensional spatial environment. Productivity and income elasticity emerge as the result of the strategic behavior of multiple agents, rather than from statistical assumptions. Moreover, Gather-Trade-Build includes trading between agents and simulates an economy over extended periods of time, i.e., spanning 10 tax periods, each of 100 days. Hence, the dynamics of Gather-Trade-Build are more complex than those considered in traditional tax frameworks and serve as a rich test bed for AI-driven policy design.

We show that the use of RL yields emergent agent behaviors that align well with economic intuition, such as specialization and tax gaming. That is, AI workers learn to specialize as traders or builders and to earn different taxable incomes over time. In particular, these phenomena are not captured through analytical approaches to tax policy design. In our setting, these learned behaviors arise due to the shape of agent and planner rewards and the economic incentives induced by the dynamics of the Gather-Trade-Build environment. This happens even with a small number of agents (4 and 10 agents in our experiments).

Note that tax gaming is not the same as tax evasion. Tax gaming refers to strategically earning different incomes at different times, while tax evasion refers to not reporting (some) earned income at all. That is, in our work, agents cannot hide their income from the planner. The literature has mainly focused on tax evasion (*17*,*18*), but not on tax gaming.

Our use of AI-driven simulations builds on agent-based modeling (ABM). Traditional ABM simulations use simulated agents with predefined behavioral rules to study emergent behavior (*19*). For instance, ABM simulations with millions of agents have analyzed systemic risk in the housing market (*20*). In settings where rational behavior may be less relevant, this approach can provide behavioral realism while avoiding problems of analytical tractability. At the same time, it also complicates the interpretation of results. Moreover, the behavior of ABM agents is often rigid and lacking in strategic or adaptive behavior, e.g., strategic responses to (a change in) economic policy.

### AI-driven policy design with two-level deep RL

The AI Economist uses two-level, deep RL to learn optimal policies: at the level of individual agents within the economy and at the level of the social planner. Both the agents and the social planner use deep neural networks to implement their policy model. Two-level RL compares the performance of billions of economic designs, making use of agents whose behaviors are learned along with the optimal planner policy.

Two-level RL is natural in many contexts, e.g., indirect mechanism design, the principal-agent problem, or regulating systems with (adversarial) agents with misaligned or unethical incentives. However, it poses a highly unstable learning problem, as agents need to continuously adapt to changing incentives. The AI Economist solves the two-level problem through the use of curriculum learning (*21*) and entropy-based regularization (*22*), providing a tractable and scalable solution.

Curriculum learning is used to start RL with easy problems (e.g., without tax) and gradually increase the difficulty (e.g., toward full income taxes). Entropy regularization adds an extra learning objective that encourages RL agents to maintain a level of randomness in their policy. Our approach stabilizes training using two key insights: (i) Agents should not face substantial utility costs that discourage exploration early during learning, and (ii) the agents and social planner should be encouraged to gradually explore and coadapt.

The AI Economist framework provides numerous advantages.

1) It does not suffer from the Lucas critique. By design, it considers actors who coadapt with economic policy.

2) Rather than behavioral rules, the use of RL provides for rational agent behavior.

3) The simulation framework is flexible, supporting a configurable number of agents and various choices in regard to economic processes.

4) The designer is free to choose any policy objective, and this does not have to be analytically tractable or differentiable.

5) RL agents can learn tabula rasa and do not strictly require prior knowledge about the structure of the economic simulation (although prior knowledge may accelerate learning). Rather, they can discover the structure of the simulation through interaction during learning.

Our work relates to computational methods for mechanism design and finding equilibria in general-sum games. Deep learning has been used to design optimal multi-item auctions, for which no analytical results are known (*12*). Moreover, evolutionary mechanism design has studied computational solutions to the planner’s problem, e.g., through iterative stepwise refinement of solutions, and satisficing constraints instead of optimization in the face of intractable complexity (*23*). However, this framework does not support rational agent behavior.

Economies with multiple agents and a social planner can also be seen as a hierarchical general-sum game, in which the planner designs payoffs for which the agents optimize their behavior. However, understanding the landscape of equilibria in general-sum games remains a substantial challenge (*4*). A computational approach to finding equilibria is empirical game-theoretic analysis (EGTA). EGTA generally constrains agent policies to a predefined set, with replicator dynamics used to find equilibria across these heuristic policies (for symmetric games with interchangeable agents and identical affordances) (*24*). Machine learning methods have been used to enhance EGTA, e.g., policy space response oracle methods where an oracle returns the best response(s) to (a set of meta-game) agent policies (*25*, *26*). However, it is hard to apply such methods to our spatiotemporal economies, which are substantially more complex than traditionally studied games and use an unbounded space of policies. Moreover, it is difficult to compute equilibria across any given set of policies, even if EGTA-style algorithms can sample from (high-dimensional) policy spaces. In contrast, using deep RL is substantially less constrained, i.e., the neural networks can implement a very high-dimensional space of policies and thus model a large and diverse set of policies.

### Optimal tax policy

We demonstrate the efficacy of the AI Economist on the problem of optimal tax policy design (*27*–*29*), which aims to improve social welfare objectives, for example, finding the right balance of equality and productivity. Specifically, we define a set of income brackets, and the AI social planner chooses the tax rates for each income bracket. Briefly, tax revenue can be used to redistribute wealth, invest in infrastructure, or fund social programs. At the same time, tax rates that are too high may disincentivize work and elicit strategic responses by taxpayers.

Theory-driven approaches to tax policy design have needed to make simplifications in the interest of analytical tractability (*30*). For example, typical models use static, one-step economies (*31*, *32*) and make use of assumptions about people’s sensitivity to tax changes (elasticity). Although work in New Dynamic Public Finance (*33*, *34*) seeks to model multistep economies, these models quickly become challenging to study analytically. Concrete results are only available for two-step economies (*35*). In stylized multistep settings, analytical methods have studied implicitly characterizations of the impact of taxes. For instance, one can derive analytical expressions for the distortion in savings or capital due to taxation (*36*), or the asymptotic behavior of taxes in the far future, the tax rate on the highest-skill agent, or the structure of incentives (*37*). However, these theoretical approaches do not yield a complete tax policy in more complex settings, such as the Gather-Trade-Build environment studied in this work. Moreover, these economic models lack interactions between agents, such as trading, and consider simple, intertemporal dynamics.

ABM simulations have also been used to study taxation, e.g., tax reporting compliance by small businesses (*38*) and designing tax audit programs that increase tax revenue with agents whose behavior was calibrated from real-world experiments (*39*). However, these works do not account for the two-level nature of policy design.

To facilitate comparison with tax policies that resemble real-world policies, we optimize tax policies within a fixed design space. For example, we assume that the RL planner only needs to learn optimal tax rates for a set of fixed income tax brackets. Hence, our learned tax policies are (approximately) optimal for that design space. However, the present framework is not limited to this choice and can handle more general tax policy design spaces.

### Experimental validation

We provide extensive proof that the AI Economist provides a sound, effective, and viable approach to understanding, evaluating, and designing economic policy design. We study optimal tax design in a single-step economy and the multistep Gather-Trade-Build environment, which implements a dynamic economy of heterogeneous, interacting agents that is more complex than the economic environments assumed in state-of-the-art tax models. We show that tax policies that are learned through two-level RL are effective, flexible, and robust to strategic agent behaviors through substantial quantitative and qualitative results:

1) In one-step economies, the AI Economist recovers the theoretically optimal tax policy derived by Saez (*31*). This demonstrates that the use of two-level RL is sound.

2) In Gather-Trade-Build economies, tax policies discovered by the AI Economist provide a substantial improvement in social welfare for two different definitions of social welfare and in various spatial world layouts; e.g., in the Open-Quadrant world with four agents, utilitarian social welfare increases by 8%, and the trade-off between equality and productivity increases by 12% over the prominent Saez tax framework (*31*).

3) In particular, AI social planners improve social welfare despite strategic behavior by AI agents seeking to lower their tax burden.

4) AI-driven tax policies improve social welfare by using different kinds of tax schedules than baseline policies from economic theory. This demonstrates that analytical methods fail to account for all of the relevant aspects of an economy, while AI techniques do not require simplifying assumptions.

5) This work also gives economic insights: It shows that the well-established Saez tax model, while optimal in a static economy, is suboptimal in dynamic economies where it fails to account for interactions between agents. Our framework enables us to precisely quantify behavioral responses and agent interactions.

## RESULTS

### AI-driven economic simulations

The AI Economist framework applies RL in two key ways: (i) to describe how rational agents respond to alternative policy choices and (ii) to optimize these policy choices in a principled economic simulation. Specifically, economic simulations need to capture the relevant economic drivers that define rational behavior. Hence, a key strength of this framework is that finding rational behaviors along with an optimal policy remains tractable even with complex specifications of economic incentives and dynamics.

#### Simulation dynamics

**Fig. 1.**
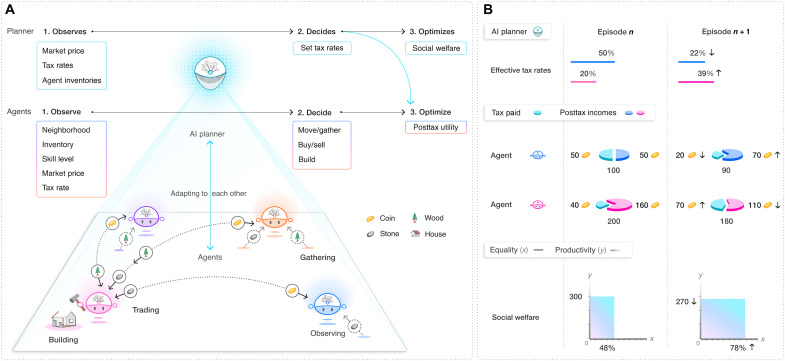
AI-driven economic simulations and two-level RL. (**A**) An AI social planner optimizes social welfare by setting income tax rates in an economic simulation with AI agents. The agents optimize their individual posttax utility by deciding how to perform labor and earn income. Both the planner and agents use RL to coadapt and optimize their behavior. Agents need to optimize behavior in a nonstationary environment, as the planner’s tax decisions change the reward that agents experience. (**B**) Illustration of coadaptation and two-level learning in an economy with two agents. Simulations proceed in episodes that last for 10 tax years, with 100 time steps in each simulated year. During learning, between any episodes *n* and *n* + 1, the planner changes tax rates, which, after behavioral changes, leads to higher social welfare, here defined as the product of productivity and equality.

We apply the AI Economist to the problem of optimal taxation (Fig. 1). The setup follows the Mirrleesian framework of nonlinear optimal taxation subject to incentive constraints (*28*). Here, the incentive constraints are represented through the rational behavior of agents, who optimize behavior subject to income tax and income redistribution.

Our simulations run for a finite number of time steps *H* and capture several key features of the Mirrleesian framework: that agents perform labor *l* to earn income *z*, where skill determines how much income an agent earns for a given amount of labor; that an agent’s utility increases with its posttax income and decreases with its labor; and that agents are heterogeneously skilled.

The simulation captures these concepts through its dynamics, i.e., the actions available to the actors and how those actions *a_t_* influence the world state *s_t_* at time step *t*. For example, agents may move spatially to collect resources, trade with one another, or spend resources to build houses; each such action accrues labor but may generate income, with higher skill ν leading to higher incomes for the same actions.

#### Taxation

Agents pay taxes on the income they earn according to a tax schedule *T*(*z*, τ), which determines taxes owed as a function of income and a set of bracketed marginal tax rates τ. The planner controls these tax rates, with all agents facing the same tax schedule, where this schedule can change at the start of each tax year. Collected taxes are evenly redistributed back to agents. For simplicity, we use fixed bracket intervals, and the planner only sets the marginal rates.

#### Behavioral models

Each actor (whether agent or planner) uses a deep neural network to encode its behavior as a probability distribution π(*a_t_* ∣ *o_t_*) over available actions, given observation *o_t_*. Following economic theory, each actor observes only a portion of the full world state *s_t_*. For instance, the planner can observe trade activity but not an agent’s skill level. Actors’ objectives, i.e., posttax utility for agents and social welfare for the planner, are captured in the reward function used to train each behavioral policy π. In this way, the AI Economist uses RL to describe rational agent behavior and optimize policy choices in complex, sequential economies beyond the reach of traditional analysis.

#### Limitations and realism

Gather-Trade-Build provides a rich test bed to show the potential of the AI Economist framework. However, it does not model the full range of economic opportunities, costs, and decisions faced by real-world individuals, nor their distribution of relevant attributes. More realistic AI-driven simulations are needed to support real-world policymaking, and defining the criteria for sufficient realism will require broad interdisciplinary design. Hence, the conclusions drawn from our experiments in Gather-Trade-Build are not meant to be applied in the real world. See the “Ethics” section for an extended discussion on ethical risk.

### Two-level RL

Under the AI Economist framework, all actors (i.e., the AI agents and the AI planner) learn and adapt using RL (algorithm S1) (*40*). Each actor learns a behavioral policy π to maximize its objective (expected sum of future rewards). Each actor also learns a value function, which estimates this expectation given observation *o_t_*. Actors iteratively explore actions by sampling from their current behavioral model and improve this model across episodes by training on experiential data. RL agents can be optimized for any reward function, and this does not have to be analytical.

An agent *i* maximizes expected total discounted utility

$$\underset{\pi_{i}}{\text{max}}E_{a_{i}∼\pi_{i},a_{-i}∼\pi_{-i},s\prime ∼P}[\sum_{t=1}^{H}\gamma^{t}r_{i,t}+u_{i,0}|\tau ],r_{i,t}=u_{i,t}-u_{i,t-1}$$ (1)

given tax rates τ, discount factor γ, and utility *u*_*i*,*t*_. Here, *s*′ is the state following *s*, and 𝒫 represents the simulation dynamics.

We use isoelastic utility (*41*)

$$u_{i,t}=\frac{C_{i,t}^{1-\eta }-1}{1-\eta }-L_{i,t},\eta >0$$ (2)

which models diminishing marginal utility over money endowment *C*_*i*,*t*_, controlled by η > 0, and the linear disutility of total labor *L*_*i*,*t*_. The planner maximizes expected social welfare

$$\underset{\pi_{p}}{\text{max}}E_{\tau ∼\pi_{p},a∼\pi ,s\prime ∼P}[\sum_{t=1}^{H}\gamma^{t}r_{p,t}+{\text{swf}}_{0}],r_{p,t}={\text{swf}}_{t}-{\text{swf}}_{t-1}$$ (3)

where swf*_t_* is social welfare at time *t*. We take swf as a utilitarian objective (an average of all agent utilities weighted by their inverse pretax income) or, alternatively, as the product of equality and productivity (representing a balance between equality and productivity). For details, see Materials and Methods.

**Fig. 2.**
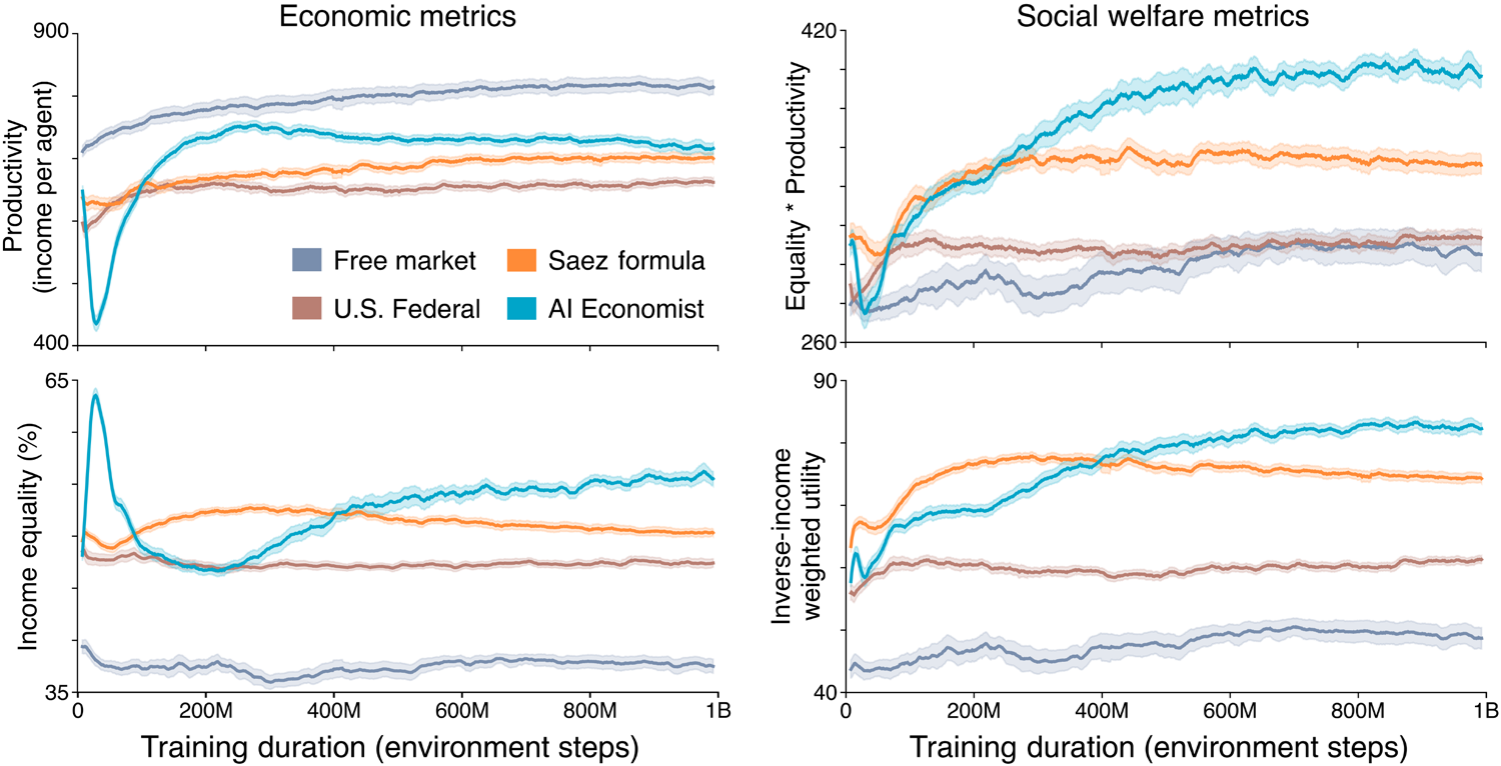
Reward achieved during training. Results are shown for 10 repetitions with different random seeds. Shading indicates the standard error. For the AI Economist (blue), the training instability due to two-level learning is clearly visible: The planner’s reward (social welfare) oscillates substantially during early training, even after using our structured curriculum learning approach.

This setup poses a two-level learning problem that features nested optimization: Agents (the inner level) need to adapt to policies set by the planner (the outer level), and vice versa (Fig. 1). This is a challenging nonstationary learning problem. For example, Fig. 2 shows that the planner’s objective does not increase monotonically when agents and planner jointly learn, even when using structured curricula and entropy regularization (which we discuss hereafter).

From an economic point of view, the planner aims to find the tax rates that maximize social welfare, subject to the best-response constraint that the behavior of agents maximizes their utility given the tax rates. From an RL point of view, a planner that learns in effect adjusts agent reward functions because taxes influence the posttax income that agents receive as a result of payments and redistributions. As the tax schedule changes, the optimal behavior for agents changes. This instability is exacerbated by mutual exploration.

### Stabilizing two-level RL

Our approach to two-level RL follows from the intuition that its stability depends on how well agents can respond to the cost of labor and (changes in) tax policies. To stabilize learning, our approach combines two key ideas: curriculum learning (*21*) and entropy regularization (*22*).

Curriculum learning starts learning with easy problems and gradually increases the difficulty. In our setting, curriculum learning effectively staggers agent and planner learning to let agents adapt to a wide range of (random) tax schedules before the planner starts to learn. In particular, we first gradually introduce labor costs and then taxes in a similar way. These curricula mitigate the key issue that suboptimal agents may incur punitively negative utility, which discourages (learning to) work. That is, agents may experience high labor costs and taxes, while earning insufficient income to yield positive utility. This may discourage RL agents from continuing to learn (given our on-policy learning approach).

Entropy regularization encourages a policy model π to maintain a level of randomness, by adding the policy entropy $-E_{\pi }[\text{log}\;\pi ]$ to the learning objective of the RL agent. We schedule the strength of entropy regularization applied to π*_p_* such that agents are initially exposed to highly random taxes. Random taxes allow agents to appropriately respond to a wide range of possible tax schedules, rather than collapsing to doing nothing. This allows the planner to optimize tax policy more stably, as taxes do not discourage agents from working. Moreover, the entropy of policies is strongly regularized to encourage exploration and gradual coadaptation between the agents and social planner throughout. For details, see Materials and Methods.

Figure 2 shows the behavior of two-level RL using our structured curricula and entropy regularization. In particular, the planner’s objective still does not increase monotonically, although eventually converging to higher social welfare than baseline tax policies. The planner’s objective decreases as the taxes are gradually enabled while its policy π*_p_* is still highly random. As the planner’s entropy coefficient anneals, its policy is no longer forced to be random. In effect, the planner begins optimizing tax rates at roughly 30 million steps, at which point its objective starts to increase. We empirically found that the planner fails to improve its objective if it starts learning prematurely, i.e., when the entropy coefficient is annealed too quickly. This is due, for example, to suboptimal agent behaviors distorting the planner’s reward signal.

### Validation in a one-step economy

The most prominent solution for optimal taxation is the analytical framework developed by Saez (*31*). This framework analyzes a simplified model where both the planner and the agents each make a single decision: The planner sets taxes, and the agents choose an amount of labor to perform. The Saez analysis describes the welfare impact of a tax rate change via its mechanical effect on redistribution and its behavioral effect on the underlying income distribution. The resulting formula computes theoretically optimal tax rates as a function of the income distribution and the elasticity of income with respect to the marginal tax rate. In practice, these income elasticities typically need to be estimated from empirical data, which is a nontrivial task (*42*).

We first validate our approach in these simplified one-step economies. Each agent chooses an amount of labor that optimizes its posttax utility. This optimal labor depends on its skill and the tax rates and does not depend on the labor choices of other agents. Before the agents act, the planner sets the marginal tax rates to optimize social welfare, taken here to be utilitarian.

We compare the economy under the Saez tax, and the AI Economist. In both cases, AI agents learn to optimize their own utility given their tax setting. The Saez tax computes tax rates based on our implementation of the Saez formula, which uses an optimal elasticity parameter found via grid search, as detailed in Materials and Methods. We include two additional baseline tax models: the free market (no taxes) and a stylized version of the U.S. Federal progressive tax schedule (see Materials and Methods for details). These baselines will be used throughout this work. There is no a priori expectation that either of the additional baselines should maximize social welfare. Rather, they provide useful comparison points and help to characterize behavioral responses to different tax policies.

**Fig. 3.**
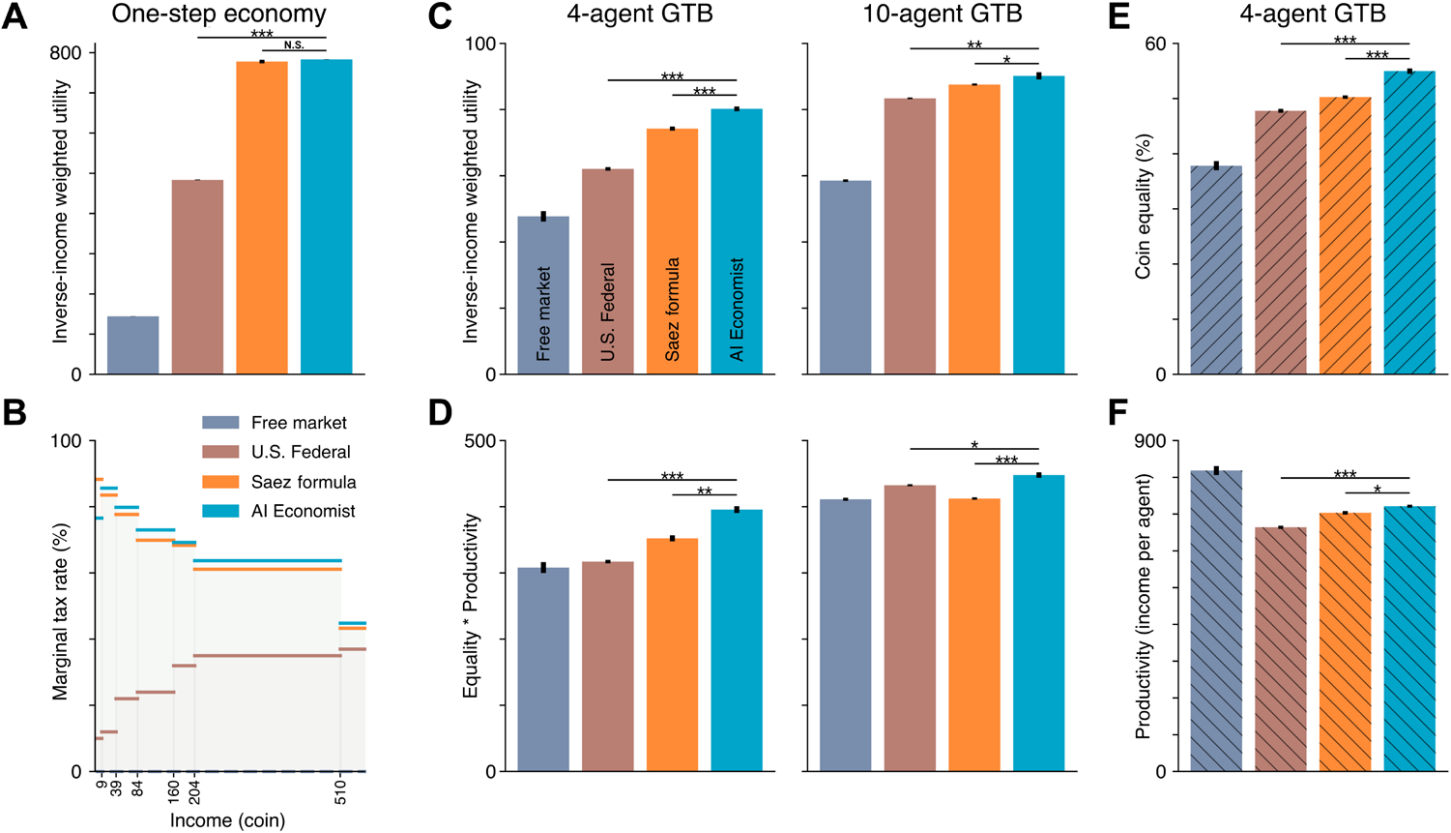
Quantitative results in a one-step economy and the Open-Quadrant Gather-Trade-Build environment. (**A** and **B**) The results of the AI Economist and the Saez tax are highly consistent in the one-step economy, in terms of both utilitarian social welfare (A) and the tax schedule (B). (**C** and **D**) In the Gather-Trade-Build environment (GTB) with 4 and 10 agents, the AI Economist outperforms baselines when optimizing the utilitarian social welfare objective (C) and when optimizing the equality-times-productivity objective (D). (**E** and **F**) Overall coin equality (E) and average productivity (F) achieved by each tax model in the four-agent Open-Quadrant scenario. Each bar represents the average end-of-training metrics over 10 random seeds (5 for the one-step economy), with error bars denoting standard error. Asterisks indicate a statistically significant difference at an α level of 0.05 (*), 0.001 (**), or 0.00001 (***). N.S. denotes not statistically significant (*P* > 0.05). All social welfare, productivity, and equality differences between the AI Economist and baselines are statistically significant, except for the difference in social welfare between the AI Economist and the Saez tax in the one-step economy (A).

The AI Economist and the Saez tax schedule produce highly consistent tax schedules and social welfare, as shown in Fig. 3 (A and B). In comparison, the free market and U.S. Federal achieve substantially worse social welfare. These results show that the AI Economist can reproduce optimal tax rates in economies that satisfy the simplifying assumptions of optimal tax theory and validate the soundness of our learning-based approach.

### Gather-Trade-Build: A dynamic economy

We study the Gather-Trade-Build economy, a two-dimensional, spatiotemporal economy with agents who move, gather resources (stone and wood), trade, and build houses. Gather-Trade-Build captures the fundamental trade-off between equality and productivity intrinsic to optimal tax design (see below) and is a rich test bed to demonstrate the advantages of AI-driven policy design. Each simulation simulates 10 tax years. Each tax year lasts 100 time steps (so that *H* = 1000), with the agents acting each time step, and the planner setting and changing tax rates at the start of each tax year. The Gather-Trade-Build environment is depicted in Fig. 1. For details, see Materials and Methods.

### Emergent phenomena in Gather-Trade-Build

A key advantage of AI-driven simulations is that they can capture macrolevel features of real economies that are emergent purely through learned rational behavior and without being manually implemented. To illustrate this, we showcase three examples of AI-driven emergent behavior in Gather-Trade-Build. Together, these examples show that AI-driven simulations capture features of real-world economies, purely through RL. Hence, AI-driven simulations provide a rich class of environments for policy design, unconstrained by analytic tractability.

#### Example 1: Emergent specialization

**Fig. 4.**
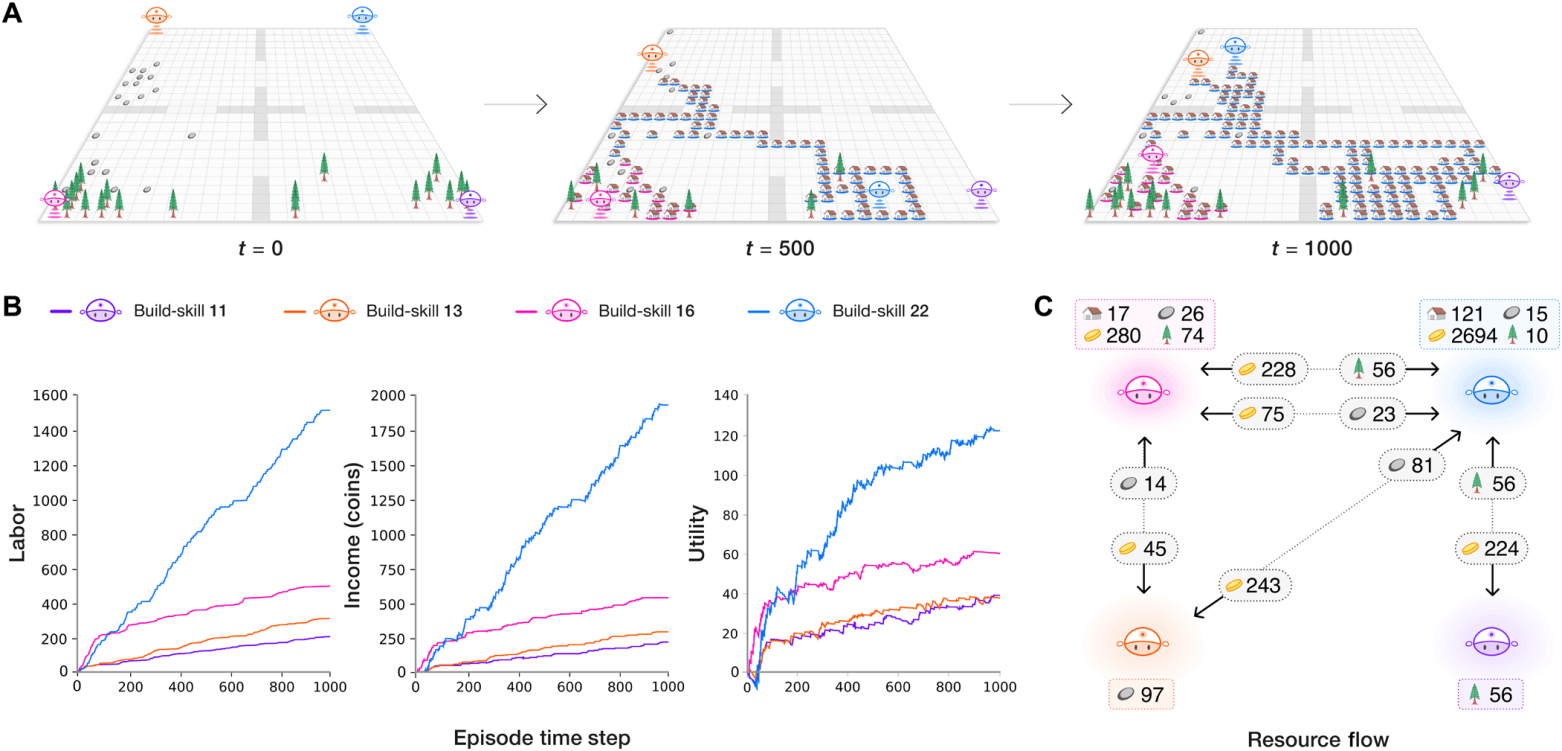
Emergent phenomena in AI-driven economic simulations under the free market. (**A**) Visualization of the spatial state of the world at *t* = 0, 500, and 1000 of an example episode in the four-agent Open-Quadrant Gather-Trade-Build scenario. Agents specialize as builders (blue agent) or gatherers (others) depending on their build-skill. (**B**) Labor, income, and utility over the course of the episode for all agents. Each quantity increases with build-skill in this setting. The highest build-skill (blue) agent chooses to do the most work and earns larger income and ultimately experience the most utility. (**C**) Net resource flow between agents during the episode. The box adjacent to each agent shows the resources it gathered and the coin it earned from building. Arrows between agents denote coin and resources exchanged through trading.

Each agent varies in its skill level. We instantiate this in our simulation as build-skill, which sets how much income an agent receives from building a house. Build-skill is distributed according to a Pareto distribution. As a result, we observe that utility-maximizing agents learn to specialize their behavior based on their build-skill (see Fig. 4). Agents with low build-skill become gatherers: They earn income by gathering and selling resources. Agents with high build-skill become builders: They learn that it is more profitable to buy resources and then build houses. This emergent behavior is entirely due to heterogeneity in skill, e.g., agents may experience different utility for different economic activity, and not due to fixed behavioral rules as in traditional ABM.

#### Example 2: Equality-productivity trade-off

**Fig. 5.**
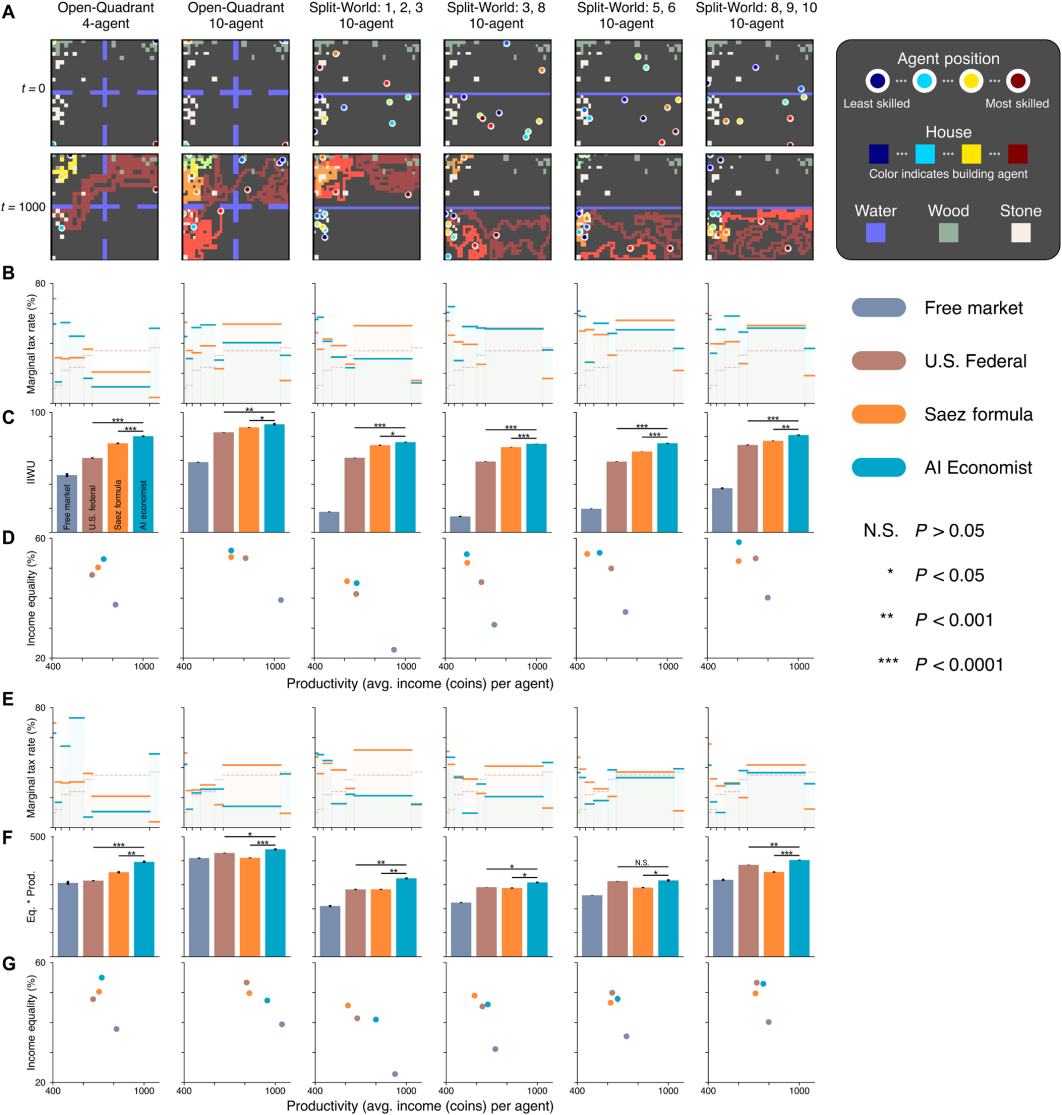
Comprehensive quantitative results in the Gather-Trade-Build environment with the utilitarian or equality-times-productivity planner objective, across all settings: Open-Quadrant and four Split-World scenarios; 4 and 10 agents. The AI Economist achieves substantially higher social welfare than all baselines. (**A**) Spatial layouts of the Open-Quadrant and Split-World scenarios at the start (*t* = 0) and end (*t* = 1000) of example episodes. (**B**) Tax schedules for the Saez tax (yellow) and the AI Economist (blue). U.S. Federal (which is the same throughout) is shown in dashed red. (**C**) Utilitarian social welfare objective (inverse-income weighted utility, labeled “IIWU”) for all planners. (**D**) Equality and productivity for all planners. For the data in (B) to (D), the AI Economist is trained to maximize the utilitarian social welfare objective, and the Saez taxes use the best-performing elasticity for the utilitarian objective. (**E** to **G**) As (B) to (D), but for the data in (E) to (G), the AI Economist is trained to maximize the equality-times-productivity social welfare objective, and the Saez taxes use the best-performing elasticity for this objective, which is shown in (F). Bars and dots represent the average end-of-training metrics over 10 (5) random seeds for the Open-Quadrant (Split-World) scenarios, with error bars denoting standard error. Asterisks indicate a statistically significant difference at an α level of 0.05 (*), 0.001 (**), or 0.00001 (***). N.S. denotes not statistically significant (*P* > 0.05). All social welfare differences between the AI Economist and baselines are statistically significant, except for the difference in equality-times-productivity (F) between the AI Economist and the U.S. Federal tax in the Split-World-5,6 scenario.

Our AI simulations capture the trade-off between equality and productivity: As tax rates increase, equality increases through wealth transfers, but productivity falls as agents are less incentivized to work due to lower posttax incomes (Figs. 3 and 5).

As a demonstration of this, the free market (no tax) baseline always yields the highest productivity and lowest equality compared to the alternative tax models. The higher productivity in the absence of taxation reflects elasticity of income with respect to taxation, and in our framework, this comes about as an emergent consequence of rational behavior.

#### Example 3: AI tax-gaming strategies

**Fig. 6.**
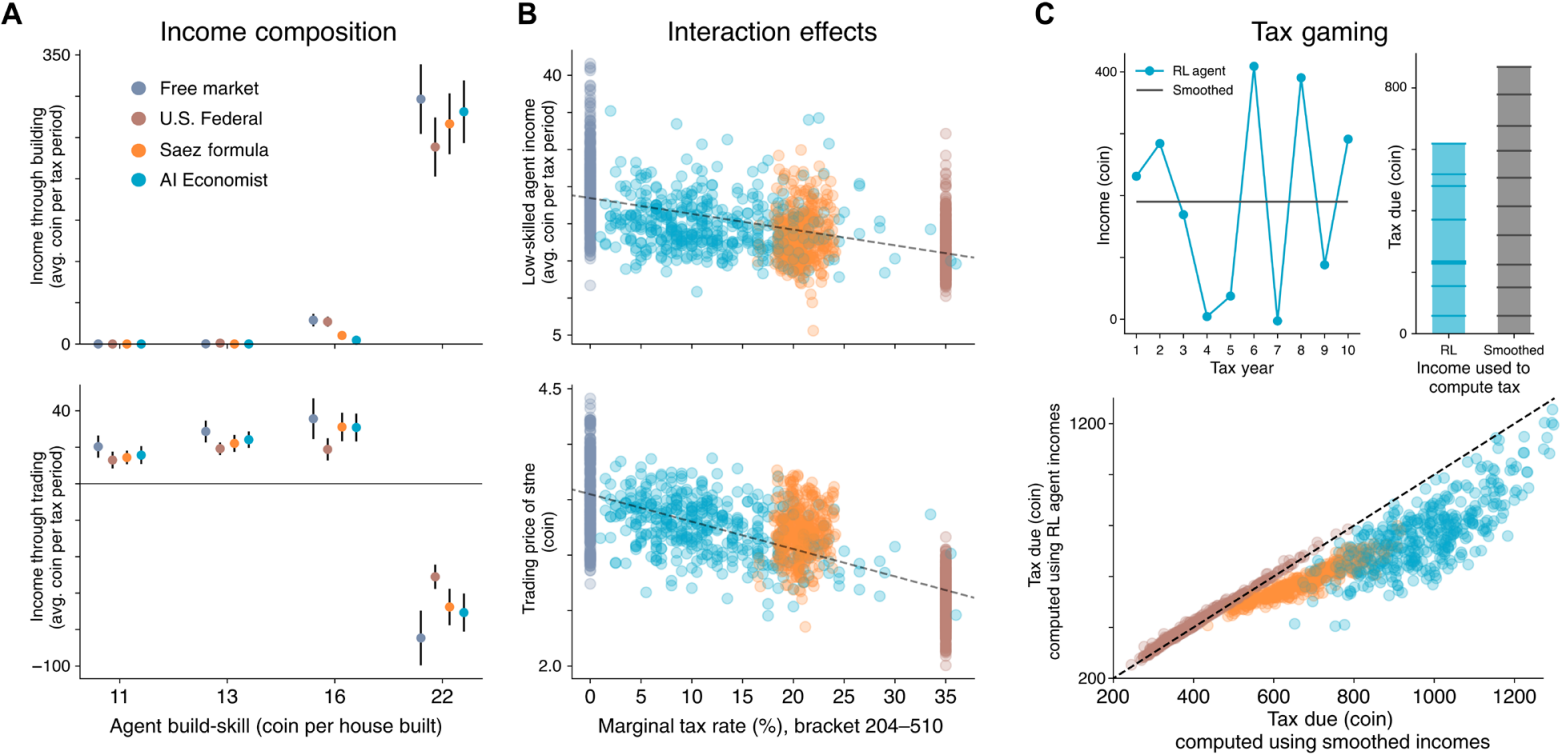
Specialization, interactions, and tax gaming in the four-agent Open-Quadrant Gather-Trade-Build environment. (**A**) Average net income from building (top) and trading (bottom) of each agent. Negative values denote net expenditure. (**B**) The income of the two lowest build-skill agents (top) and average trading price (bottom) decrease as the tax rate in the higher 204 to 510 tax bracket increases, although the agents’ incomes are below the cutoff for this bracket. Hence, the trading behavior of high-skilled agents affects the income of the low-skilled agents. The standard definition of elasticity does not capture this interaction effect. (**C**) RL agents learn to strategize across each of the 10 tax years, lowering their total payable tax compared to a smoothed strategy that earns the same average income in each year: The top panels illustrate this for a single episode; the bottom panel shows the saving relative to a smoothed income across all episodes used in the analysis. We do not observe this tax gaming under the progressive U.S. Federal tax schedule.

Our AI simulations yield emergent strategic behaviors. High-income agents learn to avoid taxes by moving labor and thus income between tax years to move more income to low-rate brackets. This can reduce the overall tax paid in comparison to earning a constant amount each year (Fig. 6C). Given the complexity of Gather-Trade-Build and similar dynamic economic environments, it is prohibitively complex for theory-driven methods to derive such temporal behavioral strategies.

### AI-driven optimal taxation

We evaluate the AI Economist across different Gather-Trade-Build economies to validate that AI-driven policy design is effective, can be applied to different economic environments, and adapts to strategic behavior more successfully than baseline tax policies.

We use two spatial layouts, Open-Quadrant and Split-World, each with different physical barrier placements and different agent starting positions. Open-Quadrant features four areas laid out in a 2 × 2 pattern, each area having a connection with its neighbor to allow agents to move between areas. Split-World features two halves, separated by an impassable water barrier. This prevents agents from moving between the top and bottom halves of the map, which blocks agents from directly accessing certain resources.

We consider four Split-World scenarios, each with 10 agents but differing in the subset of agents assigned to the resource-rich half. We consider two Open-Quadrant scenarios, with 4 agents in one version and 10 agents in the other. All six scenarios are illustrated in Fig. 5A. For ease of exposition, we focus our fine-grained analyses on results in the four-agent Open-Quadrant scenario.

#### Improved social welfare

As with the one-step economy, we compare the AI Economist against the free market, U.S. Federal, and Saez tax baselines across all of these settings (see Materials and Methods). The AI Economist achieves the highest social welfare throughout. The combined results of these experiments are presented in Fig. 5. In the Open-Quadrant layout with 4 (10) agents (Fig. 3), AI-driven taxes improve the utilitarian objective by more than 8% (2%) and the product of equality and productivity by more than 12% (8.6%) over the Saez tax.

We observe that the relative performance of the baselines depends on the choice of social welfare objective: The utilitarian objective is always higher when using the Saez tax compared to the U.S. Federal tax; however, the opposite is often true for the equality-times-productivity objective (especially in settings with 10 agents). In contrast, the AI Economist is not tailored toward a particular definition of social welfare and flexibly adjusts its tax schedule to optimize the chosen objective, yielding the best social welfare throughout. These results show the AI Economist is flexible, maintains performance with more agents, can be successfully optimized for distinct objectives, and works well in the face of adaptive, strategic behavior.

#### Similarity of learned solutions

Empirically, for a given setting, repeated RL experiments with different random seeds consistently learn a similar solution, while across settings RL converges to more dissimilar solutions.

**Fig. 7.**
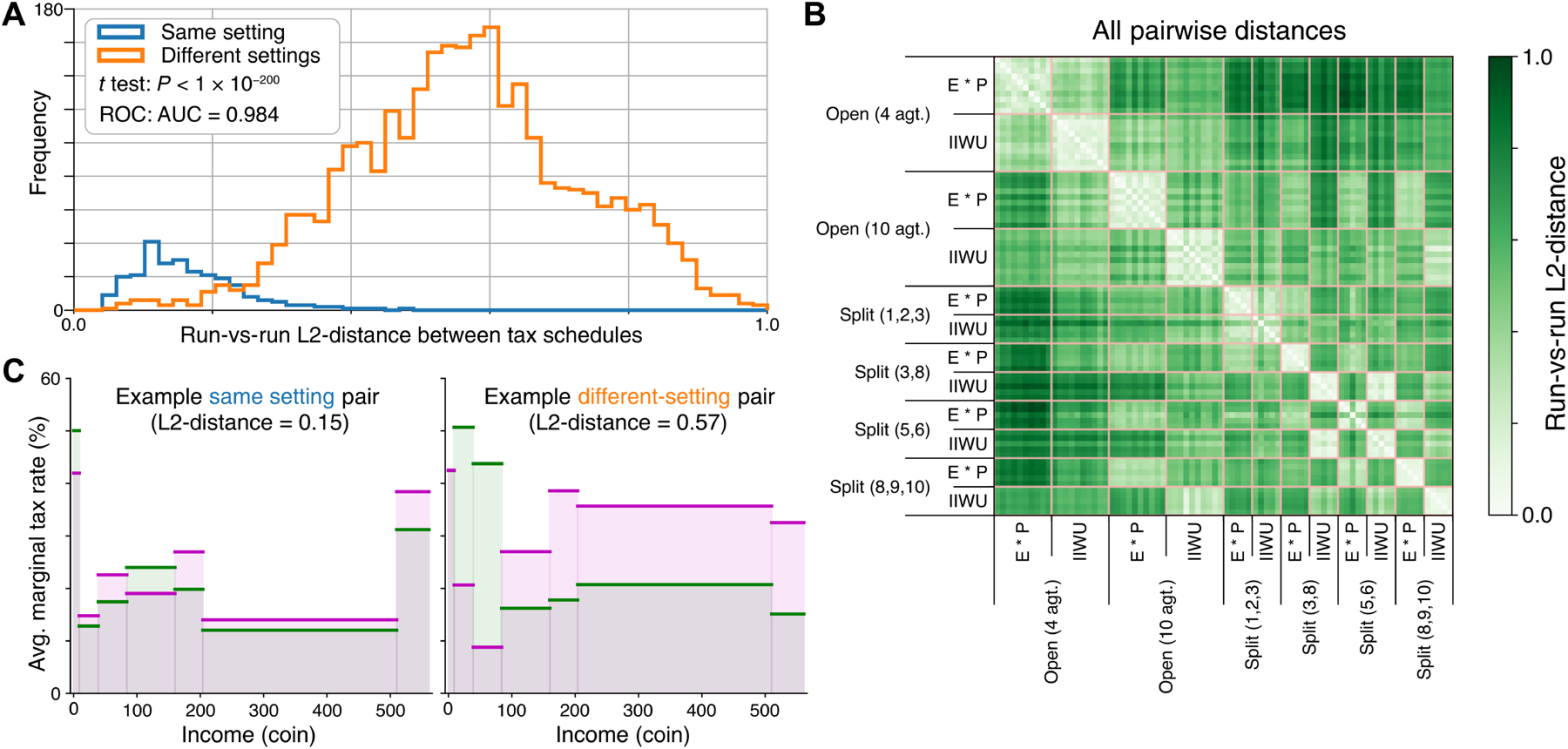
Comparing learned AI tax policies for the same and across different settings. (**A**) Distribution of L2-distances between tax schedules for each pair of distinct AI Economist runs across all 12 Gather-Trade-Build settings (each combination of the six spatial layouts and two planner objectives). Pairs from the same setting are plotted in blue, with pairs from different settings plotted in orange. Different- versus same-setting distances are statistically different (*P* < 1 × 10^−200^ under an independent *t* test), and their distributions are highly discriminable (ROC-AUC = 0.98). (**B**) Heatmap representation of the data in (A), with color intensity varying according to the L2-distance between the row and column pair of runs. The grid lines denote run indices belonging to the same setting, highlighting the block-wise diagonal structure. Note: Open-Quadrant settings have 10 runs each and Split settings have 5 runs each, which is why the grid lines and blocks are not uniformly sized. (**C**) Tax schedules for a representative same-setting pair (left) and a representative different-setting pair (right). The L2-distance of the same-setting pair approximately equals the median same-setting distance (0.15). The L2-distance of the different-setting pair approximately equals the median different-setting distance (0.57).

To quantify this, Fig. 7 visualizes instances and the distribution of L2-distances between pairs of distinct AI Economist tax policies found for distinct runs (i) in the same setting and (ii) across two different settings. There are 12 settings defined as combinations of six different instances of Gather-Trade-Build and the two different planner objectives. We find that, in the same setting, the found tax policies are highly similar (median distance = 0.15). This suggests that two-level RL converges to similar tax policies across repetitions with different random seeds. Between different settings, the found tax policies substantially differ (median distance = 0.57). This suggests that different settings lead to different learned tax policies. Different- versus same-setting distances are statistically different (means different with *P* < 10^−200^ under a *t* test), and their distributions are highly distinguishable (ROC-AUC = 0.98).

#### Adaptation during training

**Fig. 8.**
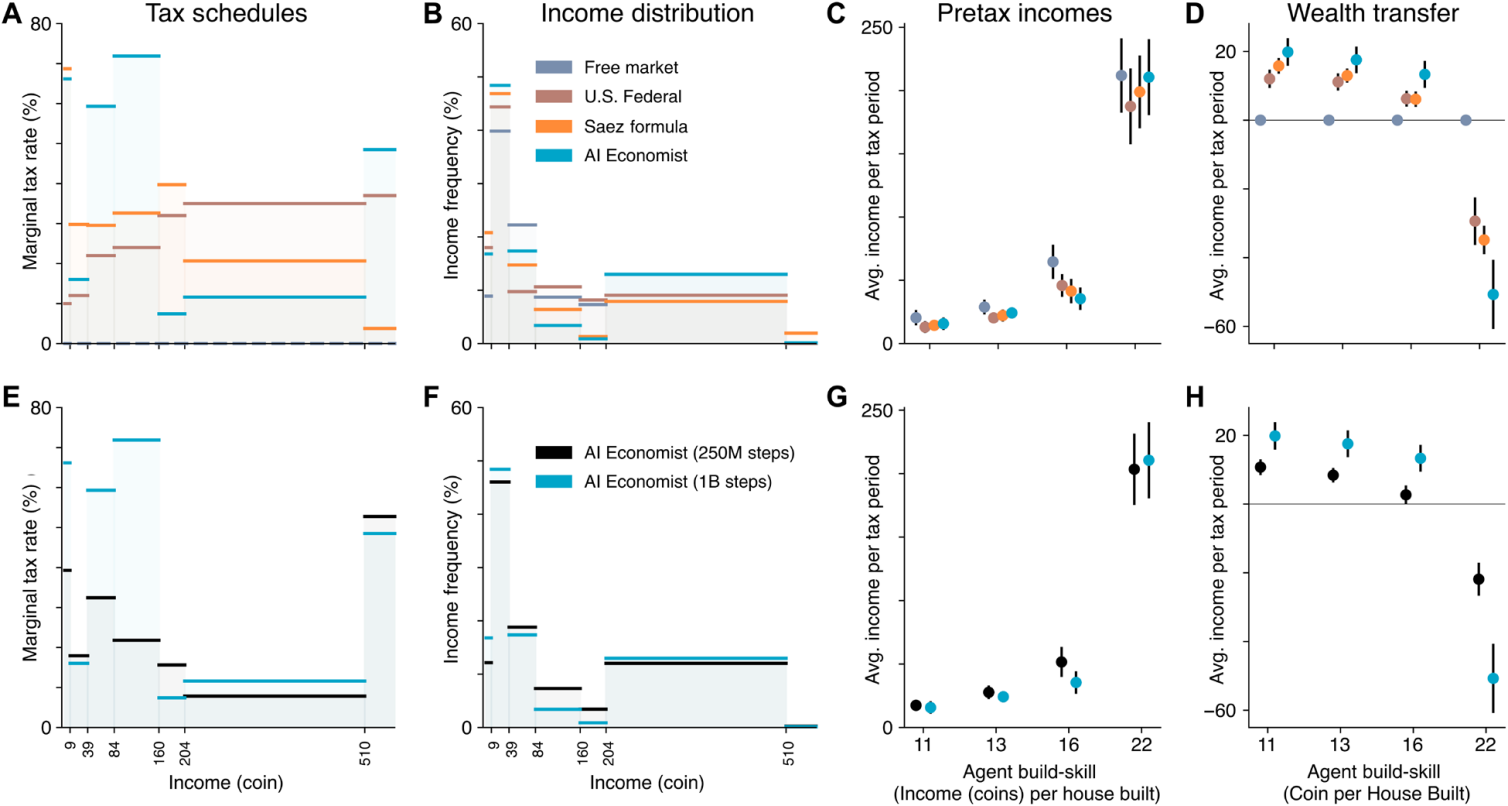
Comparison of tax policies in the four-agent Open-Quadrant Gather-Trade-Build environment. (**A**) Average marginal tax rates within each tax bracket. (**B**) Frequency with which agent incomes fall within each bracket. (**C**) Average pretax income of each agent (sorted by build-skill) under each of the tax models. (**D**) Average wealth transfer resulting from taxation and redistribution. (**E** to **H**) Same as (A) to (D), comparing the AI Economist from early during training (250 million training samples) versus at the end of training (1 billion training samples). Dots denote averages and error bars denote standard deviation across episodes.

During training, the AI Economist increases rates on the first (incomes of 0 to 9), third (39 to 84), and fourth (84 to 160) brackets, maintaining low rates otherwise (see Fig. 8). This does not substantially shift the pretax income distribution, while the posttax income distribution becomes more equal. The resulting tax schedule is distinctly different from the baselines, which use either increasing (progressive) or decreasing (regressive) schedules (Fig. 8A). The AI Economist is neither: On average, it sets the highest marginal rates for incomes between 39 and 160 coins and the lowest rates for the adjacent brackets (9 to 39 and 160 to 510 coins). Under the AI Economist, the low build-skill agents earn 9% more from trading (Fig. 6B), wealth transfers from the highest build-skill agent to others are 46% larger (Fig. 8D), income equality is at least 9% higher (Fig. 3E), and the number of incomes in the second-to-highest bracket (204 to 510 coins) is at least 64% higher and 92% smaller for the top bracket compared to baselines (Fig. 8B). These numbers are measured over the last 400 episodes within each experiment group, which amounts to 4000 total tax periods and 16,000 total incomes per group.

#### Learned AI tax policies

The AI Economist adapts to different environments: Fig. 5 shows that the best-performing AI taxes behave differently across scenarios.

For instance, in the Open-Quadrant, the AI tax schedules are similar when optimizing for the two different social welfare objectives with 4 agents but this pattern changes with 10 agents, where objective-specific tax schedules emerge. Tax rates for the brackets between 9 and 160 coins follow different patterns, for example, and overall tax rates are lower when optimizing for equality times productivity.

Furthermore, in the Split-World, the AI tax schedule depends on which agents are in the resource-rich top half of the environment. As an example, when optimizing for equality times productivity, when the two agents with the highest build-skill (agents 1, 2) are (not) in the top half, taxes in the 204 to 510 bracket are lower (higher) than those in the 0 to 84 range.

Owing to the complexity of these environments, it is not possible to provide an intuitive explanation of these AI tax schedules. It is not surprising that different scenarios can yield different optimal tax rates. For instance, agents (of varying skill) may not be able to access the same resources across different scenarios. Such differences can introduce heterogeneous economic forces and result in different learned tax policies. This is reflected even in the range of free market social outcomes across scenarios (Fig. 5, D and G). Considering that the AI tax schedules maximize social welfare within their respective scenarios, we view their scenario-specific idiosyncrasies as evidence of the adaptability of the AI Economist framework.

### Policy design beyond independence assumptions

Microfounded AI-driven simulations such as Gather-Trade-Build enable optimal tax policy design in multistep economies with coupled agent behaviors and interactions through two-level RL. In contrast, analytical solutions are not available for these kinds of environments: Traditional methods largely fail to account for interactions and thus only achieve suboptimal social welfare.

To illustrate the effect of interactions, Fig. 6 (A and B) shows that the income of the two agents with the lowest build-skill depends on the second-to-highest bracket tax rate, although this income bracket only directly applies to the agent with the highest build-skill. As this tax rate increases, the agent with the highest build-skill buys fewer resources. In turn, the average resource price as well as the trade volume decreases, reducing the incomes of the low build-skill agents. Hence, a behavioral change of one agent can change the optimal policy of another agent.

#### The effect of interactions on income elasticity and the Saez tax

However, the Saez analysis uses assumptions and a standard definition of elasticity that fail to account for interactions that arise in multistep (real-world) economies, these interactions arising through trading for example. The Saez analysis assumes that behavioral changes of agents are independent and do not affect each other. This limitation results in suboptimal policy and lost social welfare under the Saez tax, when applied to the Gather-Trade-Build environment.

To illustrate this, for the four-agent, Open-Quadrant scenario, a typical regression of observed taxes paid and reported incomes would estimate elasticity at around 0.87 (see Materials and Methods for details). However, by evaluating the Saez tax over a wide range of elasticity values, we find that an assumed elasticity of around 3 optimizes social welfare when used in Saez’s framework. Interactions between agents are a key factor for this mismatch between offline estimates and imputed optimal elasticity values.

#### The effect of interactions on tax gaming and tax revenue

Figure S1 shows the empirical relationships between trading, tax, and social metrics within and across tax policies. Trade volume is highest under AI taxes and is positively correlated with tax revenue, average income, and income equality. Tax gaming tends to increase along with tax revenue across all tax policies. The AI Economist loses more tax revenue as tax gaming increases, but its net tax revenue is still highest overall.

## DISCUSSION

The AI Economist demonstrates that economic policy design using RL, together with principled economic simulation, is sound, viable, flexible, and effective. It suggests an exciting research agenda: using AI to enable a new approach to economic design. The AI Economist framework can be used to study different policy goals and constraints and, as AI-driven simulations grow in sophistication, may help to address the modern economic divide. In particular, AI-driven simulations enable economic policies to be tested in more realistic environments than those available to analytical methods and show promise in validating assumptions in policy proposals and evaluating ideas coming from economic theory. However, these results are a first step and are not ready to be implemented as real-world policy.

### More realistic simulations

We showed that AI simulations can yield emergent behaviors that resemble the real world; for example, AI workers learn to specialize in response to economic forces. However, our simulations are not realistic in other regards, in that they lack grounding in real-world data and use a smaller number of agents than some other simulations (*20*). Whereas rational behavior, as achieved here through the use of RL, is relevant for tax policy design, it is less relevant in other settings where behavioral considerations are important (*5*). We also use relatively simple forms of agent and planner utilities and use RL to learn behaviors that optimize for these utilities.

Future research can scale up AI-driven simulations and calibrate them to real-world data, along with learning AI policies that are explainable and robust to simulation-to-reality gaps. In addition to RL training, one can collect behavioral data and constrain or calibrate policies using domain expertise to create more realistic human behavioral models and feasible government policies. Designing simulations that incorporate different societal values and are representative of different parts of society is another important direction for future work.

### Real-world feasibility of AI policies

Simplicity and explainability are also important aspects for real-world feasibility. Our results suggest that there is a trade-off between the simplicity of a tax policy and the realized social welfare. In the Gather-Trade-Build environments, AI taxes tend to blend progressive and regressive tax rates, in contrast to the Saez (regressive in our setting) and U.S. Federal (progressive) taxes. Hence, the behavior of AI taxes may be less intuitive and harder to explain, although they yield higher social welfare than simpler tax policies. Future work could further investigate and quantify the trade-off between simplicity and effectiveness of AI policies.

### Democratization through transparency

AI-driven policy design can democratize policymaking, for instance, through easily accessible open-source code releases that enable a broad multidisciplinary audience to inspect, debate, and build future policymaking frameworks. Hence, we hope that the potential of AI-driven policy design will motivate building fair and inclusive data, computation, and governance structures that ultimately improve the social good.

### Ethics

While the current version of the AI Economist provides only a limited representation of the real world, we recognize that it could be possible to manipulate future, large-scale iterations of the AI Economist to increase inequality and hide this action behind the results of an AI system. Furthermore, either out of ignorance or malice, nonrepresentative simulation environments may result in biased policy recommendations. For instance, the underrepresentation of communities and segments of the workforce in training data might lead to bias in AI-driven tax models.

Economic simulation enables studying a wide range of economic incentives and their consequences, including models of stakeholder capitalism. However, the simulation used in this work is not an actual tool that can be currently used with malintent to reconfigure tax policy.

We encourage anyone using the AI Economist to describe the ethical considerations of trained AI-driven tax models to increase transparency and trust in the system, e.g., through model cards and/or data sheets (*43*,*44*). Furthermore, we believe that any future application or policy built on economic simulations should be built on inspectable code and subject to full transparency.

To responsibly publish this research, we have taken the following measures:

1) To ensure accountability on our part, we have consulted academic experts on safe release of code and ensured that we are in compliance with their guidance. We shared the paper and an assessment of the ethical risks, mitigation strategies, and assessment of safety to publish with the following external reviewers: Dr. Simon Chesterman, Provost’s Chair and Dean of the National University of Singapore Faculty of Law, and Lofred Madzou, AI Project Lead at the World Economic Forum’s Center for the Fourth Industrial Revolution. None of the reviewers identified additional ethical concerns or mitigation strategies that should be used. All affirmed that the research is safe to publish.

2) To increase transparency, we are also publishing a summary of this work as a blog post, thereby allowing robust debate and broad multidisciplinary discussion of our work.

3) To further promote transparency, we will release an open-source version of our environment and sample training code for the simulation. This does not prevent future misuse, but we believe, at the current level of fidelity, that transparency is key to promote grounded discussion and future research.

With these mitigation strategies and other considerations in place, we believe that this research is safe to publish. Furthermore, this research was not conducted with any corporate or commercial applications in mind.

## MATERIALS AND METHODS

### One-step economy

We trained the AI Economist in a stylized, one-step economy with *N* = 100 agents, indexed by *i*, that each choose how many hours of labor *l_i_* to perform. Each agent *i* has a skill level *ν_i_*, which is a private value that represents its hourly wage. On the basis of labor, each agent *i* earns a pretax income *z_i_* = *l_i_* · *ν_i_*. Each agent *i* also pays income tax *T*(*z_i_*), which is evenly redistributed back to the agents. Hence, the posttax income is defined as ${\tilde{z}}_{i}=z_{i}-T(z_{i})+\frac{1}{N}\sum_{j=1}^{N}T(z_{j})$. As a result, each agent *i* experiences a utility $u({\tilde{z}}_{i},l_{i})={\tilde{z}}_{i}-c\cdot l_{i}^{\delta }$, which increases linearly with posttax income ${\tilde{z}}_{i}$ and decreases exponentially with labor *l_i_*, with exponent δ > 0 and constant *c* > 0 (for exact values used, see table S1).

### Gather-Trade-Build simulation

Gather-Trade-Build simulates a multistep trading economy in a two-dimensional grid-world. Table S2 provides details regarding the simulation hyperparameters. Agents can gather resources, earn coins by using the resources of stone and wood to build houses, and trade with other agents to exchange resources for coins.

Agents start at different initial locations in the world and are parameterized by different skill levels (described below). Simulations are run in episodes of 1000 time steps, which is subdivided into 10 tax periods, each lasting 100 time steps.

The state of the world is represented as an *n_h_* × *n_w_* × *n_c_* tensor, where *n_h_* and *n_w_* are the size of the world and *n_c_* is the number of unique entities that may occupy a cell, and the value of a given element indicates which entity is occupying the associated location. The action space of the agents includes four movement actions: up, down, left, and right. Agents are restricted from moving onto cells that are occupied by another agent, a water tile, or another agent’s house. Stone and wood stochastically spawn on special resource regeneration cells. Agents can gather these resources by moving to populated resource cells. After harvesting, resource cells remain empty until new resources spawn. By default, agents collect one resource unit, with the possibility of a bonus unit also being collected, the probability of which is determined by the agent’s gather-skill. Resources and coins are accounted for in each agent’s endowment *x*, which represents how many coins, stone, and wood each agent owns.

**Fig. 9.**
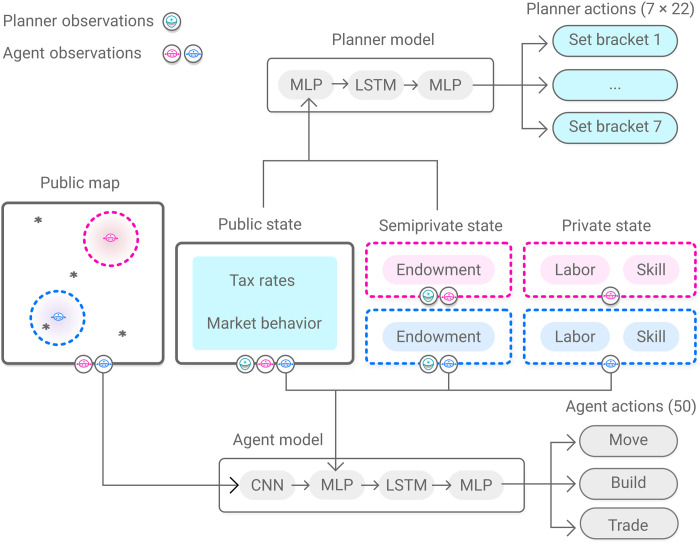
Observation and action spaces for economic agents and the social planner. The agents and the planner observe different subsets of the world state. Agents observe their spatial neighborhood, market prices, tax rates, inventories, and skill level. Agents can decide to move (and therefore gather if moving onto a resource), buy, sell, or build. There are 50 unique actions available to the agents. The planner observes market prices, tax rates, and agent inventories. The planner decides how to set tax rates, choosing one of 22 settings for each of the seven tax brackets. MLP, multilayer perceptron, LSTM, long short-term memory, CNN, convolutional neural network.

Agent observations include the state of their own endowment (wood, stone, and coin), their own skill levels, and a view of the world state tensor within an egocentric spatial window (see Fig. 9). Our experiments use a world of size 25 by 25 (40 by 40) for 4-agent (10-agent) environments, where agent spatial observations have size 11 by 11 and are padded as needed when the observation window extends beyond the world grid. The planner observations include each agent’s endowment but not skill levels (see Fig. 9). We do not include the spatial state in the planner’s observations (in pilot experiments, we observed that this choice did not affect performance).

### Trading

Agents can buy and sell resources from one another through a continuous double auction. Agents can submit asks (the number of coins they are willing to accept) or bids (how much they are willing to pay) in exchange for one unit of wood or stone. The action space of the agents includes 44 actions for trading, representing the combination of 11 price levels (0, …,10 coins), 2 directions (bids and asks), and 2 resources (wood and stone). Each trade action maps to a single order (i.e., bid three coins for one wood, ask for five coins in exchange for one stone, etc.). Once an order is submitted, it remains open until either it is matched (in which case a trade occurs) or it expires (after 50 time steps). Agents are restricted from having more than five open orders for each resource and are restricted from placing orders that they cannot complete (they cannot bid with more coins than they have and cannot submit asks for resources that they do not have). A bid/ask pair forms a valid trade if they are for the same resource and the bid price matches or exceeds the ask price. When a new order is received, it is compared against complementary orders to identify potential valid trades. When a single bid (ask) could be paired with multiple existing asks (bids), priority is given to the ask (bid) with the lowest (highest) price; in the event of ties, priority then is given to the earliest order and then at random. Once a match is identified, the trade is executed using the price of whichever order was placed first. For example, if the market receives a new bid that offers eight coins for one stone and the market has two open asks offering one stone for three coins and one stone for seven coins, received in that order, the market would pair the bid with the first ask and a trade would be executed for one stone at a price of three coins. The bidder loses three coins and gains one stone; the asker loses one stone and gains three coins. Once a bid and ask are paired and the trade is executed, both orders are removed. The state of the market is captured by the number of outstanding bids and asks at each price level for each resource. Agents observe these counts for both their own bids/asks and the cumulative bids/asks of other agents. The planner observes the cumulative bids/asks of all agents. In addition, both the agents and the planner observe historical information from the market: the average trading price for each resource, as well as the number of trades at each price level.

### Building

Agents can choose to spend one unit of wood and one unit of stone to build a house, and this places a house tile at the agent’s current location and earns the agent some number of coins. Agents are restricted from building on source cells as well as locations where a house already exists. The number of coins earned per house is identical to an agent’s build-skill, a numeric value between 10 and 30. Hence, agents can earn between 10 and 30 coins per house built. Skill is heterogeneous across agents and does not change during an episode. Each agent’s action space includes one action for building.

### Labor

Over the course of an episode of 1000 time steps, agents accumulate labor cost, which reflects the amount of effort associated with their actions. Each type of action (moving, gathering, trading, and building) is associated with a specific labor cost. All agents experience the same labor costs.

### Taxation mechanism

Taxation is implemented using income brackets and bracket tax rates. All taxation is anonymous: Tax rates and brackets do not depend on the identity of taxpayers. The payable tax for income *z* is computed as follows

$$T(z)=\sum_{j=1}^{B}\tau_{j}\cdot ((b_{j+1}-b_{j})1[z>b_{j+1}]+(z-b_{j})1[b_{j}<z\leq b_{j+1}])$$ (4)

where *B* is the number of brackets, and τ*_j_* and *b_j_* are marginal tax rates and income boundaries of the brackets, respectively.

Each simulation episode has 10 tax years. On the first time step of each tax year, marginal tax rates are set that will be used to collect taxes when the tax year ends.

For baseline models, tax rates are set either formulaically or fixed. For taxes controlled by a deep neural network, the action space of the planner is divided into seven action subspaces, one for each tax bracket: (0,0.05,0.10, …,1.0)^7^. Each subspace denotes the set of discretized marginal tax rates available to the planner. Discretization of tax rates only applies to deep learning networks, enabling standard techniques for RL with discrete actions. We emphasize that the income bracket cutoffs are fixed.

Each agent observes the current tax rates, indicators of the temporal progress of the current tax year, and the set of sorted and anonymized incomes the agents reported in the previous tax year. In addition to this global tax information, each agent also observes the marginal rate at the level of income it has earned within the current tax year so far. The planner also observes this global tax information, as well as the non-anonymized incomes and marginal tax rate (at these incomes) of each agent in the previous tax year.

### Redistribution mechanism

An agent’s pretax income *z_i_* for a given tax year is defined simply as the change in its coin endowment *C_i_* since the start of the year. Accordingly, taxes are collected at the end of each tax year by subtracting *T*(*z_i_*) from *C_i_*.

Taxes are used to redistribute wealth: The total tax revenue is evenly redistributed back to the agents. In total, at the end of each tax year, the coin endowment for agent *i* changes according to $\Delta C_{i}=-T(z_{i})+\frac{1}{N}\sum_{j=1}^{N}T(z_{j})$, where *N* is the number of agents. Through this mechanism, agents may gain coin when they receive more through redistribution than they pay in taxes.

### Gather-Trade-Build scenarios

We considered two spatial layouts: Open-Quadrant and Split-World (see Fig. 5).

Open-Quadrant features four regions delineated by impassable water with passageways connecting each quadrant. Quadrants contain different combinations of resources: both stone and wood, only stone, only wood, or nothing. Agents can freely access all quadrants, if not blocked by objects or other agents.

Split-World features two disconnected regions: The top contains stone and wood, while the bottom only has stone. Water tiles prevent agents from moving from one region to the other.

All scenarios use a fixed set of build-skills based on a clipped Pareto distribution (sampled skills are clipped to the maximum skill value) and determine each agent’s starting location based on its assigned build-skill. The Open-Quadrant scenario assigns agents to a particular corner of the map, with similarly skilled agents being placed in the same starting quadrant. (Agents in the lowest build-skill quartile start in the wood quadrant; those in the second quartile start in the stone quadrant; those in the third quartile start in the quadrant with both resources; and agents in the highest build-skill quartile start in the empty quadrant.) The Split-World scenario allows control over which agents have access to both wood and stone versus access to only stone. We consider four Split-World variations, each with 10 agents. Each variation gives stone and wood access to a specific subset of the 10 agents, as determined by their build-skill rank. For example: Split-World-1,2,3 places the three highest-skilled agents in the top, Split-World-8,9,10 places the three lowest-skilled agents in the top, and Split-World-5,6 places the two middle-skilled agents in the top.

### Agent utility

Following optimal taxation theory, agent utilities depend positively on accumulated coin *C*_*i*,*t*_, which only depends on posttax income $\tilde{z}=z-T(z)$. In contrast, the utility for agent *i* depends negatively on accumulated labor $L_{i,t}=\sum_{k=0}^{t}l_{i,k}$ at time step *t*. The utility for an agent *i* is

$$u_{i,t}=\frac{C_{i,t}^{1-\eta }-1}{1-\eta }-L_{i,t}$$ (5)

Agents learn behaviors that maximize their expected total discounted utility for an episode. We found that build-skill is a substantial determinant of behavior; agents’ gather-skill empirically does not affect optimal behavior in our settings.

All of our experiments use a fixed set of build-skills, which, along with labor costs, are roughly calibrated so that (i) agents need to be strategic in how they choose to earn income and (ii) the shape of the resulting income distribution roughly matches that of the 2018 U.S. economy with trained optimal agent behaviors.

### Social planner

The simulation environment includes a social planner who uses tax policy and lump-sum redistribution to influence social outcomes. Each episode is divided into 10 tax years. At the start of each tax year, the planner chooses a tax schedule *T*(*z*) that determines the amount of taxes each agent will owe as a function of its income *z* earned during the tax year and redistributes tax revenue.

We compare four kinds of planners: (i) Free market: a fixed-rate planner where all tax rates are 0%; (ii) U.S. Federal: a fixed-rate planner where bracketed marginal tax rates follow a progressive scheme adapted from the 2018 U.S. Federal single-filer income tax schedule; (iii) Saez tax: an adaptive planner that computes theoretically optimal marginal rates using the empirical income distribution and elasticity of income with respect to taxation; and (iv) AI Economist: a deep neural network, adaptive planner that maps a set of planner observations to bracketed marginal tax rates, which is trained via RL to maximize social welfare.

### Two-level deep RL

RL provides a flexible way to simultaneously optimize and model the behavioral effects of tax policies. We instantiate RL at two levels, that is, for two types of actors: training agent behavioral policy models and a taxation policy model for the social planner.

We train each actor’s behavioral policy using deep RL, which learns the weights θ*_i_* of a neural network π(*a*_*i*, *t*_∣*o*_*i*,*t*_; θ*_i_*) that maps an actor’s observations to actions. Network weights are trained to maximize the expected total discounted reward of the output actions.

Specifically, for an agent *i* using a behavioral policy π*_i_*(*a_t_*∣*o_t_*; θ*_i_*), the RL training objective is (omitting the tax policy π*_p_*)

$$\underset{\pi_{i}}{\text{max}}E_{a_{1}∼\pi_{1},\ldots ,a_{N}∼\pi_{N},s\prime ∼P}[\sum_{t=0}^{H}\gamma^{t}r_{t}]$$ (6)

where *s*′ is the next state and 𝒫 denotes the dynamics of the environment. The objective for the planner policy π*_p_* is similar. Standard model-free policy gradient methods update the policy weights θ*_i_* using (variations of)

$$\Delta \theta_{i}\propto E_{a_{1}∼\pi_{1},\ldots ,a_{N}∼\pi_{N},s\prime ∼P}[\sum_{t=0}^{H}\gamma^{t}r_{t}\nabla_{\theta_{i}}\text{log}\;\pi_{i}(a_{i,t}|o_{i,t};\theta_{i})]$$ (7)

In our work, we use proximal policy gradients (PPO) (*45*), an extension of Eq. 7 to train all actors (both agents and planner).

To improve learning efficiency, we train a single-agent policy network π(*a*_*i*,*t*_∣*o*_*i*,*t*_; θ) whose weights are shared by all agents, that is, θ*_i_* = θ. This network is still able to embed diverse, agent-specific behaviors by conditioning on agent-specific observations.

At each time step *t*, each agent observes the following: its nearby spatial surroundings; its current endowment (stone, wood, and coin); private characteristics, such as its building skill; the state of the markets for trading resources; and a description of the current tax rates. These observations form the inputs to the policy network, which uses a combination of convolutional, fully connected, and recurrent layers to represent spatial, nonspatial, and historical information, respectively. For recurrent components, each agent maintains its own hidden state. This is visualized in Fig. 9. For the detailed model architecture and training hyperparameters, see tables S3 and S4.

The policy network for the social planner follows a similar construction but differs somewhat in the information it observes. Specifically, at each time step, the planner policy observes the following: the current inventories of each agent, the state of the resource markets, and a description of the current tax rates. The planner cannot directly observe private information such as an agent’s skill level.

### Training objectives

Rational economic agents train their policy π*_i_* to optimize their total discounted utility over time while experiencing tax rates τ set by the planner’s policy π*_p_*. The agent training objective is

$$\forall i;\underset{\pi_{i}}{\text{max}}E_{\tau ∼\pi_{p},a_{i}∼\pi_{i},a_{-i}∼\pi_{-i},s\prime ∼P}[\sum_{t=1}^{H}\gamma^{t}r_{i,t}+u_{i,0}],\;r_{i,t}=u_{i,t}-u_{i,t-1}$$ (8)

where the instantaneous reward *r*_*i*,*t*_ is the marginal utility for agent *i* at time step *t*, and we use the isoelastic utility *u_t_* as defined in Eq. 5. Bold-faced quantities denote vectors, and the subscript “−*i*” denotes quantities for all agents except for *i*.

For an agent population with monetary endowments ***C****_t_* = (*C*_1,*t*_, …, *C*_*N*,*t*_), we define equality eq(***C****_t_*) as

$$\text{eq}(C_{t})=1-\frac{N}{N-1}\text{gini}(C_{t}),\;0\leq \text{eq}(C_{t})\leq 1$$ (9)

where the Gini index is defined as

$$\text{gini}(C_{t})=\frac{\sum_{i=1}^{N}\sum_{j=1}^{N}|C_{i,t}-C_{j,t}|}{2N\sum_{i=1}^{N}C_{i,t}},\;0\leq \text{gini}(C_{t})\leq \frac{N-1}{N}$$ (10)

We also define productivity as the sum of all incomes

$$\text{prod}(C_{t})=\sum_{i}C_{i,t}$$ (11)

Note that we assume that the economy is closed: Subsidies are always redistributed evenly among agents, and no tax money leaves the system. Hence, the sum of pretax and posttax incomes is the same. The planner trains its policy π*_p_* to optimize social welfare

$$\underset{\pi_{p}}{\text{max}}E_{\tau ∼\pi_{p},a∼\pi ,s\prime ∼P}[\sum_{t=1}^{H}\gamma^{t}r_{p,t}+{\text{swf}}_{0}],r_{p,t}={\text{swf}}_{t}-{\text{swf}}_{t-1}$$ (12)

The utilitarian social welfare objective is the family of linear-weighted sums of agent utilities, defined for weights ω*_i_* ≥ 0

$${\text{swf}}_{t}=\sum_{i=1}^{N}\omega_{i}\cdot u_{i,t}$$ (13)

We use inverse-income as the weights: $\omega_{i}\propto \frac{1}{C_{i}}$, normalized to sum to 1. We also adopt an objective that optimizes a trade-off between equality and productivity, defined as the product of equality and productivity

$${\text{swf}}_{t}=\text{eq}(C_{t})\cdot \text{prod}(C_{t})$$ (14)

### Hyperparameter settings

Our multiagent RL framework features both environment and training hyperparameters that are relevant to experiments.

We tuned the environment hyperparameters to roughly align the income distribution under U.S. Federal taxes (in the four-agent case) with income frequencies reported under those taxes in 2018. The main drivers of both the income distribution and specialization in Gather-Trade-Build are agents’ build-skill (the only activity that directly generates income) and their access to resources. Hence, the environment hyperparameters influence the income distribution by influencing the heterogeneity in initial locations and build-skill. In addition to parameters of the agents’ skill distributions, we tuned the labor costs and the convexity parameter η of the agents’ utility function.

We tuned planner and agent learning rates via grid search. Hyperparameters related to structured curricula (see below) were similarly tuned using a combination of grid and manual search. All other hyperparameters, e.g., related to network structure, batch size, and training parallelism, were chosen to yield reasonable training throughput and stability.

The discount factor γ influences how “forward looking” the optimal policy is, i.e., the relative priority of near- versus long-term rewards. Our experiments use discount factor γ = 0.998 throughout. This choice reflects the practical benefits of setting γ < 1 but was not otherwise tuned based on training performance. For reference, when using γ = 0.998, the relative weight of future rewards decays to about 0.37 after $\frac{1}{1-\gamma }=500$ steps, with episodes lasting 1000 steps.

### Training strategies

Two-level RL can be unstable, as the planner’s actions (setting tax rates) affect agent rewards (marginal utility depending on posttax income).

We use three learning curricula and two training phases to stabilize two-level RL. In phase one, agent policies are trained from scratch in a free-market (no-tax) environment for 50 million steps. In phase two, agents continue to learn in the presence of taxes for another 1 billion steps.

The first learning curriculum occurs during phase one: Agents use a curriculum in phase one that anneals the utility cost associated with labor. The reason is that many actions cost labor, but few yield income. Hence, if exploring without a curriculum, a suboptimal policy can experience too much labor cost and converge to doing nothing.

The second learning curriculum occurs during phase two: We anneal the maximum marginal tax to prevent planners from setting extremely high taxes during exploration that reduce posttax income to zero and discourage agents from improving their behaviors.

We also carefully balance entropy regularization to prevent agent and planner policies from prematurely converging and promote the coadaption of agent and planner policies. The entropy of the policy π for agent *i*, given an observation *o_i_*, is defined as

$$\text{entropy}(\pi )=-E_{a∼\pi }[\text{log}\;\pi (a|o_{i};\theta_{i})]$$ (15)

When training the AI Economist planner, we introduce the third learning curriculum by annealing the level of planner policy entropy regularization. Enforcing highly entropic planner policies during the early portion of phase two allows the agents to learn appropriate responses to a wide range of tax levels before the planner is able to optimize its policy.

### Training procedure

For training, we use PPO on mini-batches of experience collected from 30 parallel replicas of the simulation environment. Each environment replica runs for 200 steps during a training iteration. Hence, for each training iteration, 6000 transitions are sampled for the planner and *N*· 6000 transitions are sampled for the agents, where *N* is the number of agents in the scenario, using the latest policy parameters.

The planner policy model is updated using transition mini-batches of size 1500, with one PPO update per mini-batch (four updates per iteration). The agent policy model is updated using transition mini-batches of size 400 (1500) for 4-agent (10-agent) scenarios (40 updates per iteration). Table S4 provides details regarding the training hyperparameters. Algorithm S1 describes the full training procedure. These can be found in the Supplementary Materials.

### Action spaces and masks

Both agents and planners use discrete action spaces. We use action masking to prevent invalid actions, e.g., when agents cannot move across water, and to implement learning curricula. Masks control which actions can be sampled at a given time by assigning zero probability to restricted actions.

In addition, we include a no-operation action (NO-OP) in each action space. For the planner, each of the seven action subspaces includes a NO-OP action. The NO-OP action allows agents to idle and the planner to leave a bracket’s tax rates unchanged between periods.

Action masks allow the planner to observe every time step while only acting at the start of each new tax year. After the first time step of a tax year, action masks enforce that only NO-OP planner actions are sampled.

### Saez tax

The Saez tax computes tax rates using an analytical formula (*31*) for a one-step economy with income distribution *f*(*z*) and cumulative distribution *F*(*z*). These rates maximize a weighted average ∑*_i_w_i_u_i_* of agent utilities, where the weights *w_i_* reflect the redistributive preferences of the planner, and are optimal in an idealized one-step economy. The Saez tax computes marginal rates as

$$\tau (z)=\frac{1-G(z)}{1-G(z)+a(z)e(z)}$$ (16)

where *z* is pretax income, *G*(*z*) is an income-dependent social welfare weight, and *a*(*z*) is the local Pareto parameter.

Specifically, let α(*z*) denote the marginal average income at income *z*, normalized by the fraction of incomes above *z*, i.e.,

$$\alpha (z)=\frac{z\cdot f(z)}{1-F(z)}$$ (17)

Let *G*(*z*) denote the normalized, reverse cumulative Pareto weight over incomes above a threshold *z*, i.e.,

$$G(z)=\frac{1}{1-F(z)}\int_{z\prime =z}^{\infty }p(z\prime )g(z\prime )dz\prime$$ (18)

where *g*(*z*) is the normalized social marginal welfare weight of an agent earning income *z*, and 1 − *F*(*z*) is the fraction of incomes above income *z*. In this way, *G*(*z*) represents how much the social welfare function weights the incomes above *z*. Let elasticity *e*(*z*) denote the sensitivity of an agent’s income to changes in the tax rate when that agent’s income is *z*, defined as

$$e(z)=\frac{1-\tau (z)}{z}\frac{\mathrm{dz}}{d(1-\tau (z))}$$ (19)

Both *G*(*z*) and *a*(*z*) can be computed directly from the (empirical) income distribution but typically *e*(*z*) needs to be estimated (which is challenging). We set the social welfare weights $w_{i}\propto \frac{1}{z_{i}}$, normalized so the sum over all individuals is 1. This choice encodes a welfare focus on low-income agents.

### Empirical income distribution and Saez tax rates

To apply the Saez tax, we use rollout data from a temporal window of episodes to estimate the empirical income distribution and compute *G*(*z*) and *a*(*z*). We aggregate reported incomes over a look-back window. We maintain a buffer of recent incomes reported by the agents, where each data point in this buffer represents the income reported by a single agent during a single tax year. Each simulation episode includes 10 tax years. Hence, a single agent may report incomes in multiple different brackets in a single episode.

To compute *G*(*z*) and *a*(*z*), we first discretize the empirical income distribution and compute τ(*z*) within each of the resulting income bins. To get the average tax rate τ for each tax bracket, we take the average of the binned rates over the bracket’s income interval. Following the Saez analysis (*31*), when computing the top bracket rate, *G*(*z*) is the total social welfare weight of the incomes in the top bracket, and *a*(*z*) is computed as $\frac{m}{m-z^{+}}$, where *m* is the average income of those in the top bracket, and *z*^+^ is the income cutoff for the top bracket (510 in our implementation; see Fig. 8).

The empirical income distribution in Gather-Trade-Build is a clipped Pareto distribution; a true Pareto distribution has a fatter tail. This leads to a regressive Saez tax schedule. More generally, the Saez tax can yield both regressive or progressive policy, based on the shape of the income distribution. The two common cases discussed in the literature are Pareto and log-normal income distributions, which yield progressive and regressive tax schedules, respectively. Hence, Saez taxes under a clipped Pareto income distribution more closely resemble those under a log-normal income distribution.

### Estimating elasticity

The most substantial obstacle to implementing the Saez tax is correctly identifying the elasticity *e*(*z*), defined as in Eq. 19. Owing to the complexity of the Gather-Trade-Build economy and agent learning dynamics, it is challenging to reliably measure local elasticities *e*(*z*) as a function of income *z*. The large variance in empirical incomes caused large variance in the estimated local elasticity, leading to unstable two-level RL.

Therefore, we used a global elasticity estimate *e*, which assumes that elasticity is the same at all income levels. Empirically, we observe that the elasticity does not vary greatly across income ranges, hence justifying using a global elasticity.

**Fig. 10.**
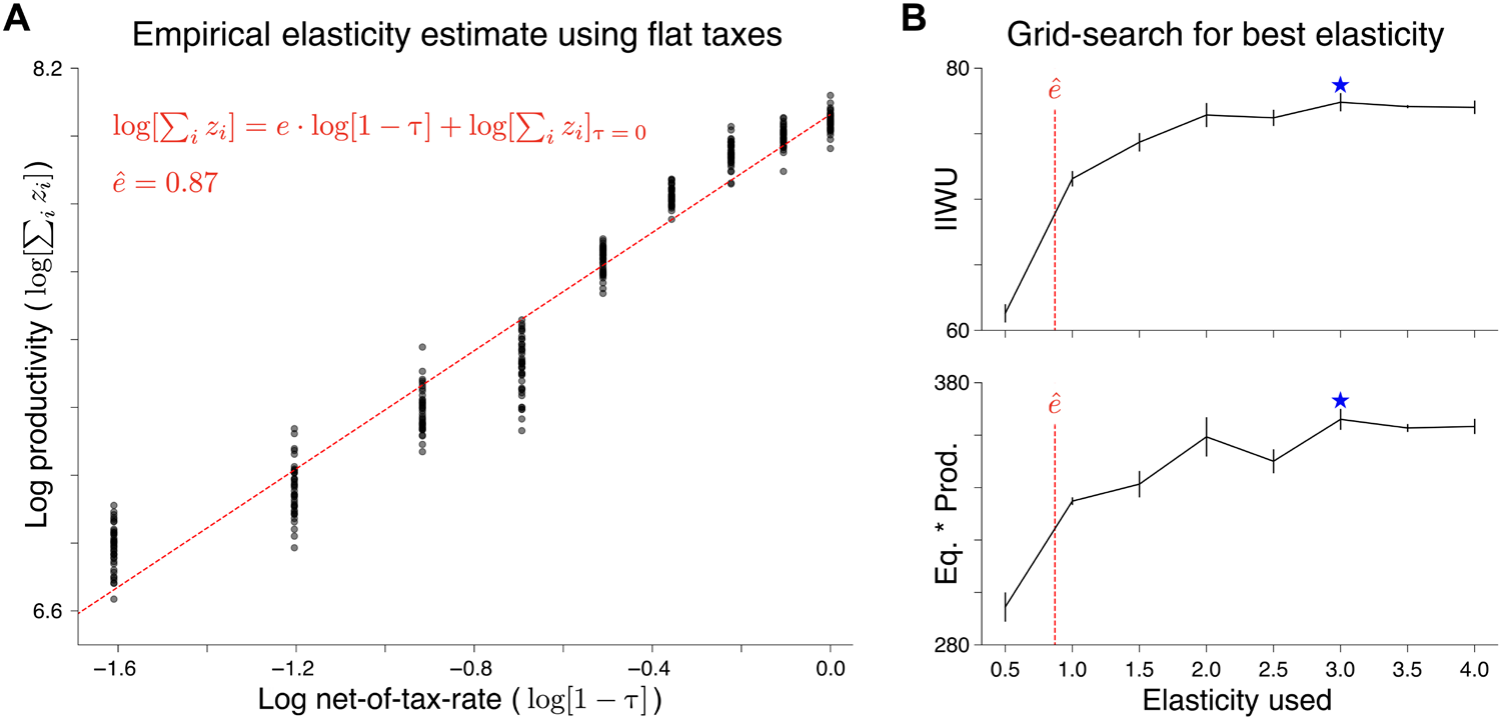
Estimating elasticity for the Saez tax in the four-agent Open-Quadrant scenario. (**A**) Regression on income and marginal tax rate data yields elasticity estimates $\hat{e}$ of approximately 0.87 (slope of the red dotted line). The net-of-tax-rate (1 − τ) is the fraction of income agents retain after paying taxes. Productivity (∑*_i_z_i_*) is the total pretax income earned by the agents. Each dot represents a (∑*_i_z_i_*, τ) pair observed from a sweep over flat tax rates (see Materials and Methods). (**B**) Social welfare with agents trained to convergence under the Saez tax, using a grid-search over elasticity parameters. Social welfare is highest under the Saez tax when the used elasticity parameter is approximately 3 (blue star), for both the inverse-income-weighted-utility objective (top) and the equality-times-productivity objective (bottom). Error bars denote standard error across the three random seeds used for each elasticity value.

For comparison, we also estimated the elasticity *e*(*z*) using classic techniques, which use regression on observed incomes and marginal tax rates obtained from agents trained under varying fixed flat-tax systems (*42*). Using a global constant elasticity for all agents, we instantiate this method by regression on *K* tuples ${\left[(Z_{k},\tau_{k})\right]}_{k=1}^{K}$ of observed total income *Z* = ∑*_i_z_i_* and manually fixed flat tax rates τ in the simulation. Specifically, we use a linear model

$$\text{log}\;(Z)=\hat{e}\cdot \text{log}\;(1-\tau )+\text{log}\;({\hat{Z}}^{0})$$ (20)

where ${\hat{Z}}^{0}$ is a bias unit. Using a flat tax rate ensures that agents always face the same tax rate during episodes, allowing for more consistent estimates. To generate data, we sweep over a range of values for τ and collect observed total income data *Z*. This yields an estimate of $\hat{e}∼1$, which produces suboptimal social welfare (see Fig. 10).

To provide the best possible performance for the Saez framework, we optimize the Saez tax using a grid search over possible *e* values. For each scenario, we separately conduct experiments involving sweeps over a range of potential values of *e* and select the best-performing one for each social welfare objective to use as a fixed elasticity estimate. This yields an optimal elasticity estimate of *e* ∼ 3 in the four-agent Open-Quadrant scenario, substantially higher than that estimated through regression techniques (see Fig. 10).

### Quantification and statistical significance

All experiments in the Open-Quadrant Gather-Trade-Build scenarios were repeated with 10 random seeds; experiments in the Split-World Gather-Trade-Build scenarios and the One-Step Economy were repeated with five random seeds.

For a given repetition, we compute each performance metric, e.g., equality or social welfare, as its average value over the last 3000 episodes of training (the last 100 episodes for each of the 30 parallel environments). We report the average and standard error of these metrics across the 5 or 10 random seeds within a particular experiment group (Figs. 3 and 5). Statistical significance is computed using a two-sample *t* test.

In other analyses (Figs. 6 and 8), we compute agent-wise statistics, e.g., pretax income and wealth transfer, using agent-specific statistics for each of the 10 tax periods in the episode. We conduct our analyses using the 40 most recent episodes (before the end of training, or before 250 million training steps where noted) for each repetition. For these analyses, we report the averages and standard deviations across the 400 associated episodes within each group of experiments.

## Supplementary Materials

### This PDF file includes:

Tables S1 to S4 Algorithm S1 Fig. S1
