# Supplementary material for: The AI Economist: Taxation policy design via two-level deep multiagent reinforcement learning
Source: Sci Adv. 2022 May 4;8(18):eabk2607. doi: 10.1126/sciadv.abk2607 (PMC9067926; doi:10.1126/sciadv.abk2607)
Supplement: Supplementary file 1 — Tables S1 to S4 Algorithm S1 Fig. S1 [file sciadv.abk2607_sm.pdf]

Science Advances
AAAS

Supplementary Materials for

The AI Economist: Taxation policy design via two-level deep multiagent reinforcement learning

Stephan Zheng*, Alexander Trott, Sunil Srinivasa, David C. Parkes, Richard Socher

*Corresponding author. Email: st.t.zheng@gmail.com

Published 4 May 2022, *Sci. Adv.* **8**, eabk2607 (2022)
DOI: 10.1126/sciadv.abk2607

This PDF file includes:

Tables S1 to S4
Algorithm S1
Fig. S1

Parameter		Value
Number of agents	N	100
Minimum skill value		1.24
Maximum skill value		159.1
Maximum labor choice		100
Labor disutility coefficient	c	0.0005
Labor disutility exponent	δ	3.5
Min bracket rate		0%
Max bracket rate		100%
Rate discretization (AI Economist)		5%

Table S1: Hyperparameters for the One-Step Economy environment.

Parameter		Value
Episode length	H	1000
World height	n_h	25 (4-agent); 40 (10-agent)
World width	n_w	25 (4-agent); 40 (10-agent)
Resource respawn probability		0.01
Max resource health		1
Starting agent coin	$C_{i,0}$	0
Iso-elastic utility exponent	η	0.23
Move labor		0.21
Gather labor		0.21
Trade labor		0.05
Build labor		2.1
Minimum build payout		10
Build payment max skill multiplier		3
Max bid/ask price		10
Max bid/ask order duration		50
Max number of open orders per resource		5
Tax period duration	$\mathcal{T}$	100
Min bracket rate		0%
Max bracket rate		100%
Rate discretization (AI Economist)		5%

Table S2: Hyperparameters for the Gather-Trade-Build environment.

Parameter		Value
Number of parallel environment replicas		30
Sampling horizon (steps per replica)	$\mathcal{H}$	200
Agent SGD minibatch size (# agents = 4)		600
Agent SGD minibatch size (# agents = 10)		1500
Planner SGD minibatch size		1500
SGD sequence length		25
Policy updates per horizon (agent)		40
Policy updates per horizon (planner)		4
CPUs		15
Learning rate (agent)		0.0003
Learning rate (planner)		0.0001
Entropy regularization coefficient (agent)		0.025
Entropy regularization coefficient (planner)		0.125
Discount factor	γ	0.998
Gradient clipping norm threshold		10
Value function loss coefficient		0.05
Phase *one* training duration		25M steps
Phase *two* training duration		1B steps
Phase *two* initial max τ		10%
Phase *two* tax annealing duration		27M steps
Phase *two* entropy regularization annealing duration		50M steps

Table S3: Hyperparameters for two-level reinforcement learning (RL), which trains multiple agents and a social planner. The base RL algorithm is the proximal policy gradient algorithm (*45*).

Parameter	Value
Number of convolutional layers	2
Number of fully-connected layers	2
Fully-connected layer dimension (agent)	128
Fully-connected layer dimension (planner)	256
LSTM cell size (agent)	128
LSTM cell size (planner)	256
Agent spatial observation box half-width	5

Table S4: Hyperparameters for the neural networks implementing the agent and planner policy models.

Algorithm S1 Two-level Reinforcement Learning. Agents and social planner learn simultaneously. Bold-faced symbols indicate quantities for multiple agents. Note that agents share weights.

Input

$\mathcal{H}$ Sampling horizon
$\mathcal{T}$ Tax period length
$\mathbb{A}$ On-policy learning algorithm (in this work, PPO (*45*))
$\mathcal{C}$ Stopping criterion (for instance, agent and planner rewards have not improved)

Output

θ Trained agent policy weights
ϕ Trained planner policy weights

$s, \boldsymbol{o}, o_p, \boldsymbol{h}, h_p \Leftarrow s_0, \boldsymbol{o}_0, o_{p,0}, \boldsymbol{h}_0, h_{p,0}$ ▷ Reset episode: initialize world state s, observation o, hidden states h
$\theta, \phi \Leftarrow \theta_0, \phi_0$ ▷ Initial agent and planner policy weights
$\mathcal{D}, \mathcal{D}_p \Leftarrow \{\}, \{\}$ ▷ Reset agent and planner transition buffers
while training **do**
    for $t = 1, \ldots, \mathcal{H}$ **do**
        $\boldsymbol{a}, \boldsymbol{h} \Leftarrow \boldsymbol{\pi}(\cdot|\boldsymbol{o}, \boldsymbol{h}, \theta)$ ▷ Sample agent actions; update hidden state
        if t mod $\mathcal{T}$ = 0 **then** ▷ First timestep of tax period
            $\tau, h_p \Leftarrow \pi_p(\cdot|o_p, h_p, \phi)$ ▷ Sample marginal tax rates; update planner hidden state
        else
            $\texttt{no-op}, h_p \Leftarrow \pi_p(\cdot|o_p, h_p, \phi)$ ▷ Only update planner hidden state
        end if
        $s', \boldsymbol{o}', o'_p, \boldsymbol{r}, r_p \Leftarrow \texttt{Env.step}(s, \boldsymbol{a}, \tau)$ ▷ Next state / observations, pre-tax reward, planner reward
        if t mod $\mathcal{T}$ = $\mathcal{T}$ - 1 **then** ▷ Last timestep of tax period
            $s', \boldsymbol{o}', o'_p, \boldsymbol{r}, r_p \Leftarrow \texttt{Env.tax}(s', \tau)$ ▷ Apply taxes; compute post-tax rewards
        end if
        $\mathcal{D} \Leftarrow \mathcal{D} \cup \{(\boldsymbol{o}, \boldsymbol{a}, \boldsymbol{r}, \boldsymbol{o}')\}$ ▷ Update agent transition buffer
        $\mathcal{D}_p \Leftarrow \mathcal{D}_p \cup \{(o_p, \tau, r_p, o'_p)\}$ ▷ Update planner transition buffer
        $s, \boldsymbol{o}, o_p \Leftarrow s', \boldsymbol{o}', o'_p$
    end for
    Update θ, ϕ using data in $\mathcal{D}, \mathcal{D}_p$ and $\mathbb{A}$.
    $\mathcal{D}, \mathcal{D}_p \Leftarrow \{\}, \{\}$ ▷ Reset agent and planner transition buffers
    if episode is completed **then**
        $s, \boldsymbol{o}, o_p, \boldsymbol{h}, h_p \Leftarrow s_0, \boldsymbol{o}_0, o_{p,0}, \boldsymbol{h}_0, h_{p,0}$ ▷ Reset episode
    end if
    if criterion $\mathcal{C}$ is met **then return** θ, ϕ
    end if
end while

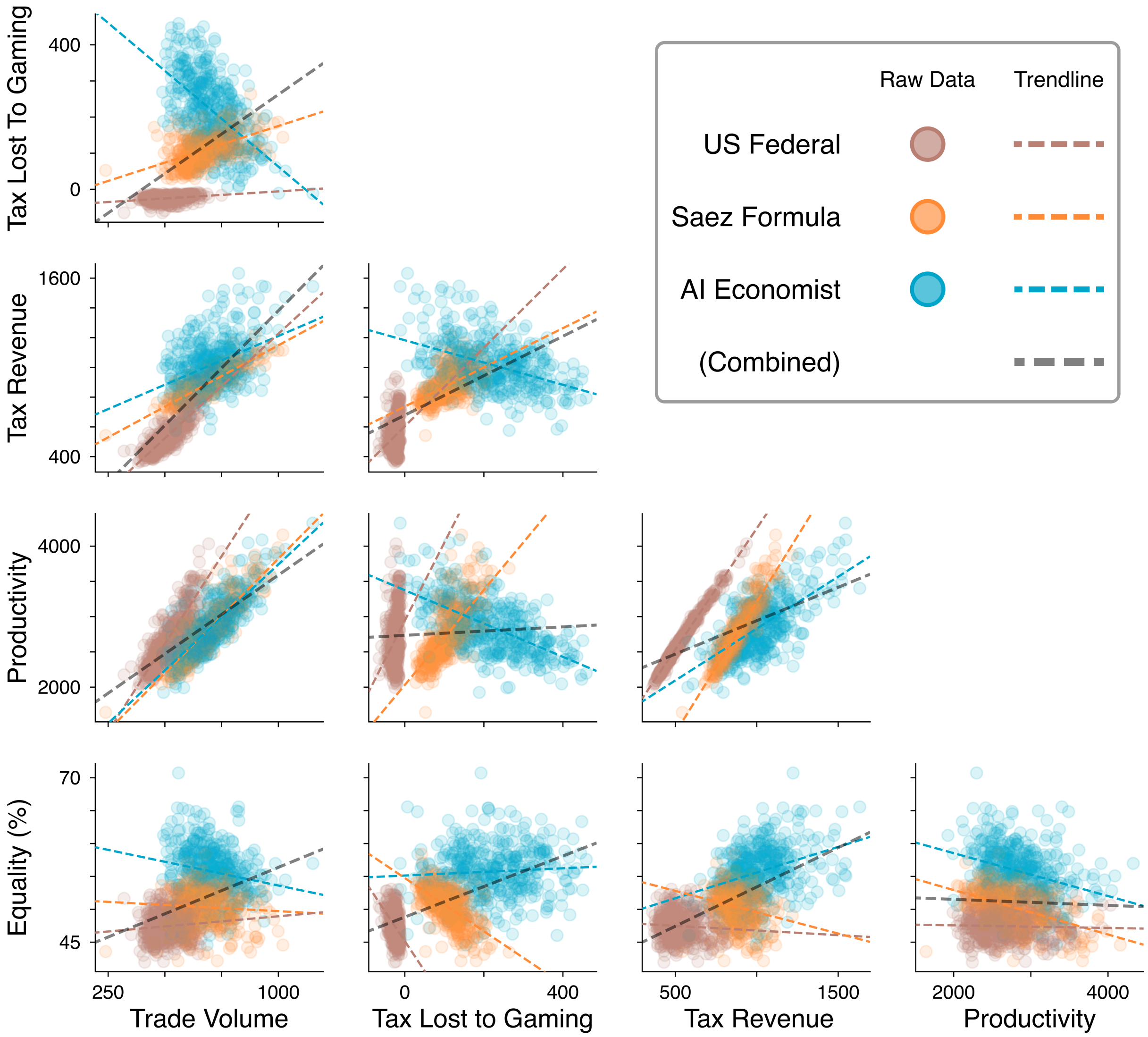

Figure S1: **Trading and economic performance, including tax revenue, in the 4-agent Open-Quadrant Gather-Trade-Build environment.** The scatter plots show the empirical relationships between trading, tax gaming, tax revenue, productivity, and equality. Each dot represents a single simulation episode in the 4-agent, Open Quadrant setting. Colored dashed lines provide trend lines (via linear regression) for the data with the matching color. The gray dashed line provides an aggregate trend line for the combined data. All metrics (except for equality) are measured in Coins. *Trade Volume* measures the total (positive) income through trading. *Tax Lost to Gaming* measures the difference between the total tax revenue and the hypothetical tax revenue if there were no temporal variation in incomes. *Tax Revenue* measures the total taxes collected during the episode. *Productivity* measures the total income generated during the episode. *Equality* measures the equality of post-tax incomes, where 100% equals perfect equality.
